# Enhancing Sn-Pb perovskite homogeneity via thioether coordination for efficient and stable all-perovskite tandem solar cells

**DOI:** 10.1126/sciadv.aeb8790

**Published:** 2026-07-10

**Authors:** Lijuan He, Haoran Wang, Zhaojin Wang, Zhongliang Yan, Arui Huang, Jing Zhang, Xueying Yang, Miao Zeng, Zaiwei Wang, Guang Yang, Zhifang Shi, Wei Zhang, Liang Shen, Yang Bai, Hui-Ming Cheng

**Affiliations:** ^1^ Institute of Technology for Carbon Neutrality, Shenzhen Institutes of Advanced Technology, Chinese Academy of Sciences, Shenzhen 518055, China.; ^2^ Faculty of Materials Science and Energy Engineering, Shenzhen University of Advanced Technology, Shenzhen 518107, China.; ^3^ Advanced Technology Institute (ATI), University of Surrey, Guildford, Surrey GU2 7XH, United Kingdom.; ^4^ School of Materials Science and Engineering, Zhengzhou University, Zhengzhou 450001, China.; ^5^ Department of Electrical and Electronic Engineering, Photonic Research Institute (PRI), Research Institute of Smart Energy (RISE), Research Institute for Advanced Manufacturing (RIAM), The Hong Kong Polytechnic University, Hung Hom, Kowloon, Hong Kong, China.; ^6^ State Key Laboratory of Integrated Optoelectronics, College of Electronic Science and Engineering, Jilin University, Changchun 130012, China.; ^7^ Shenyang National Laboratory for Materials Sciences, Institute of Metal Research, Chinese Academy of Sciences, Shenyang 110016, China.

## Abstract

All-perovskite tandem solar cells (TSCs) hold a substantial promise for achieving ultrahigh-efficiency photovoltaics beyond the Shockley-Queisser limit. However, their development has been hampered by challenges associated with a narrow-bandgap tin-lead (Sn-Pb) perovskite subcell. A key issue is inhomogeneous Sn/Pb distribution during crystallization, which generates trap states and accelerates degradation. Here, we introduce a molecular stabilization strategy by using *S*-allyl-l-cysteine (SALC) as a ligand that preferentially coordinates with tin(II) iodide (SnI_2_), thereby modulating crystallization kinetics. The strong thioether coordination leads to spatially uniform Sn/Pb distribution, and 3.5-fold reduction in Sn(IV) content due to the reduction capability of functional groups in SALC. Consequently, the resulting Sn-Pb perovskite solar cells achieve a champion power conversion efficiency (PCE) of 22.99% with an exceptional open-circuit voltage of 0.892 V. When integrated into all-perovskite TSCs, a certified PCE of 28.84% (29.44% laboratory-measured) is achieved along with a great improvement in operational stability compared to control devices, retaining nearly 90% of initial PCE after 420 hours of the maximum power point tracking under 1 sun illumination in ambient air.

## Teaser

A sulfur-containing additive stabilizes tin-lead perovskites, enabling efficient and durable all-perovskite tandem solar cells.

## INTRODUCTION

The emergence of perovskite solar cells (PSCs) has revolutionized photovoltaic research, with a certified power conversion efficiency (PCE) exceeding 27% for single-junction devices (*1*). All-perovskite tandem solar cells (TSCs), which combine wide-bandgap (WBG; ~1.7 to 1.9 eV) and narrow-bandgap (NBG; ~1.2 to 1.3 eV) subcells, offer a promising route to surpass the Shockley-Queisser limit of single-junction devices (*2*–*13*). Despite the fact that the certified efficiency of all-perovskite TSCs have recently exceeded 30% (*14*), these values still fall considerably behind their theoretical potential of 45% and their operational stability is far from the requirement for commercialization. A key issue that leads to such performance discrepancy is the inferior optoelectronic quality of tin-lead (Sn-Pb) NBG perovskite absorbers, where inhomogeneous Sn/Pb distribution during crystallization not only creates electronic defects but also accelerates Sn^2+^ oxidation—a critical yet overlooked relationship that lies at the heart of both efficiency and stability challenges in these materials (*15*).

The crystallization dynamics of Sn-Pb perovskites are governed by competing kinetic and thermodynamic factors. Sn^2+^ exhibits higher electrophilicity due to the higher energy of its outermost 5p orbitals, resulting in stronger Lewis acidity than Pb^2+^ (*16*). Second, SnI_2_ has a lower solubility in *N*,*N*′-dimethylformamide (DMF)/dimethyl sulfoxide (DMSO) solvents that causes undesirable crystal precipitation during film formation (*17*). Consequently, SnI_2_ demonstrates higher reactivity with organic components [formamidinium iodide (FAI)] and a faster nucleation kinetics than lead iodide (PbI_2_), leading to inhomogeneity and phase segregation during film formation (*18*–*22*). This inhomogeneous distribution induces detrimental defects and damages stability in Sn-Pb perovskite films (*23*–*25*). Third, compared to Pb, the absence of lanthanide contraction in Sn leads to low ionization energy and electron affinity in tin compounds. Therefore, Sn^2+^ exhibits a much shallower thermodynamic sink than Pb^2+^, leading to higher susceptibility to oxidation and poorer thermodynamic stability.

Previous attempts to stabilize Sn-Pb perovskites include composition regulation (*26*–*28*), interfacial modification (*29*–*35*), and additive engineering (*7*, *13*, *36*–*46*). Although reducing agents like tin fluoride (SnF_2_) (*38*) and antioxidant additives (*7*, *40*, *41*) can temporarily suppress Sn^2+^ oxidation, they fail to prevent the inherent inhomogeneity that drives instability. Conventional ammonium salts and thiocyanate salts are able to modulate crystallization kinetics and enhance the film quality but lack binding selectivity required for achieving Sn/Pb homogeneity (*42*–*45*). These strategies fail to address the fundamental limitations of NBG perovskite devices: Sn/Pb inhomogeneity, Sn oxidation issues, and uncontrolled crystallization kinetics, which ultimately govern their stability and efficiency.

Here, we demonstrate that rational ligand design enabling homogeneous Sn/Pb distribution represents a feasible strategy in stabilizing Sn-Pb perovskites and all-perovskite TSCs. We reveal that *S*-allyl-l-cysteine (SALC) as a multifunctional modulator that simultaneously addresses two critical challenges in Sn-Pb perovskites: (i) strong Sn-coordination agent from thioether and carboxyl groups with Sn^2+^/Pb^2+^, retarding the perovskite nucleation rate, leading to congruent crystallization and homogeneous Sn/Pb spatial distribution; (ii) suppressed Sn^2+^ oxidation is achieved through a dual mechanism: excellent passivation provided by all functional groups (thioether, carboxyl, and amino groups) collectively and elevated intrinsic oxidation barrier. As a result, SALC-modified Sn-Pb PSCs yield a champion PCE of 22.99%, with an open-circuit voltage (*V*_OC_) deficit of merely 0.358 V, representing one of the lowest reported values for Sn-Pb compositions used in TSCs (table S1). When incorporated into all-perovskite tandems, we achieve a high efficiency of 29.44% without encapsulation (certified 28.84%). The encapsulated tandem device retains nearly 90% initial PCE after 420 hours of the maximum power point tracking (MPPT) in ambient air, representing a large improvement in stability compared to control devices.

## RESULTS

### Ligand design for Sn/Pb homogeneity

The rapid crystallization of tin has been demonstrated to lead to inhomogeneity in the Sn-Pb composition (*47*–*50*), which may lead to electronic disorder. We leverage the strong coordination capability of sulfur-containing groups with metallic ions (*51*, *52*), which can occupy the vacant orbitals of Sn^2+^ to form robust Sn-S coordination, thereby suppressing Sn^2+^ oxidation and stabilizing the perovskite lattice. Complementary amino (─NH_2_) and carboxyl (─COOH) functionalities are anticipated to synergistically stabilize Sn-Pb perovskites: the amino groups through hydrogen bonding with iodide ions (I^−^), and the carboxyl groups through electrostatic interactions with Sn^2+^/Pb^2+^, thereby suppressing oxidative degradation. l-Cysteine (LC), a naturally occurring amino acid with demonstrated metal-coordinating capability, commonly used in stabilizing gold nanoparticles, served as a foundational ligand scaffold for Sn-Pb perovskites. However, considering the inherent susceptibility of its ─SH groups to oxidation (forming disulfide bonds), we introduce electron-delocalizing π-conjugated moieties—a phenyl ring and C═C bond—to modify the sulfur environment, transforming it into a more stable thioether configuration in *S*-benzyl-l-cysteine (SBLC) and SALC while enhancing the molecular coordination capacity.

**Fig. 1.**
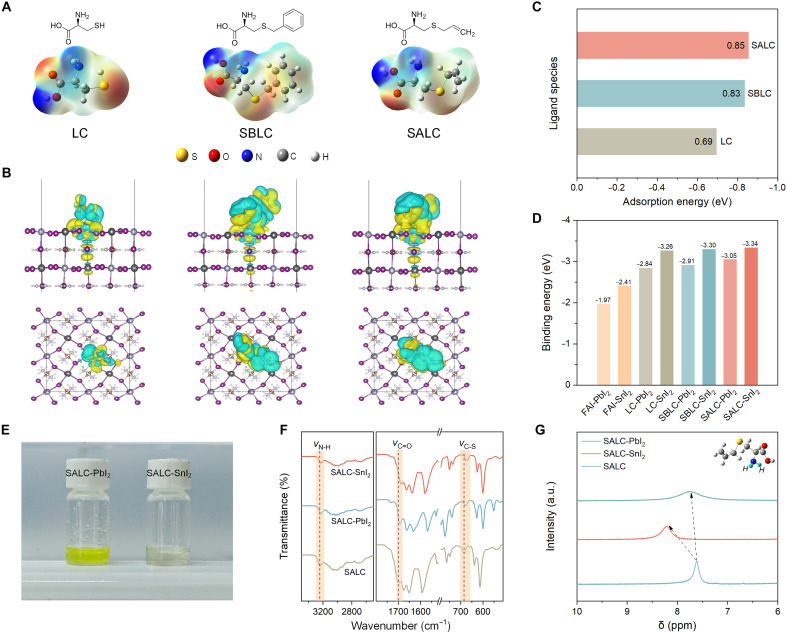
DFT calculation and chemistry studies. (**A**) Structure and ESP of LC, SBLC, and SALC ligands. (**B**) Differential charge density distribution of the optimized atomic structures of ligands absorbed on the perovskite surfaces, side view (above) and top view (below). (**C**) Histogram of adsorption energies of LC, SBLC, and SALC ligands absorbed on the perovskite surfaces. (**D**) The binding energies of FAI-PbI_2_, FAI-SnI_2_, LC-PbI_2_, LC-SnI_2_, SBLC-PbI_2_, SBLC-SnI_2_, SALC-PbI_2_, and SALC-SnI_2_ complexes. (**E**) Photographs of SALC-PbI_2_ and SALC-SnI_2_ dissolved in DMF/DMSO mixed solvents (0.1 M). (**F**) FTIR spectra of the SALC, SnI_2_/PbI_2_ mixed with SALC. (**G**) ^1^H NMR spectra of the SALC, SALC with SnI_2_ or PbI_2_. a.u., arbitrary units.

We carried out theoretical calculations to reveal the structure-function relationships among LC, SBLC, and SALC. As shown in Fig. 1A, electrostatic potential (ESP) analysis demonstrates that π-conjugated phenyl and C═C groups in SBLC/SALC reduce the electronegativity of sulfur atoms by dispersing electron density via π-electron delocalization compared to LC (*53*, *54*). The rigid conjugated structure of phenyl introduces steric hindrance, slightly weakening Sn^2+^/Pb^2+^ coordination. The C═C bond’s π-electrons delocalize to adjacent sulfur atoms through the conjugated framework, moderately lowering the electron density of sulfur while enhancing whole molecular electron delocalization. This dual effect enhances molecular stability without compromising coordination strength. Furthermore, the conjugated structures enhance the electronegativity of the carboxyl groups (─COOH), strengthening its coordination with Sn^2+^/Pb^2+^ and electrostatic interaction with surface iodide ions (I^−^). Flexible C═C chains exhibit superior conformational adaptability compared to rigid phenyl rings, which may enable optimized molecular alignment with local surface configurations of the perovskite crystals (pending further experiments). The increased dipole moment further validated that the ligand design enhances adsorption energetics and strengthens interactions with Sn-Pb perovskite (fig. S1) (*55*, *56*). Density functional theory (DFT) calculations reveal the optimized adsorption configurations of three ligands on the Sn-Pb perovskite surface (fig. S2). Differential charge density distribution analyses further visualize the ligand-perovskite interactions, as illustrated in Fig. 1B. Distinct differences in charge density are observed among the three adsorption configurations: the SALC-perovskite systems exhibit more pronounced electron accumulation (cyan regions), elucidating the strong interactions between SALC and Sn-Pb perovskite (*57*). This conclusion was corroborated by the higher adsorption energy (Fig. 1C). The introduction of C═C bonds optimized the electron cloud density of the ligand molecule, stabilizing the adsorption conformation and enhancing the adsorption energy. ESP analysis revealed that the introduction of a conjugated moiety amplifies the localized positive charge density on the amino group, thereby promoting stronger hydrogen-bonding interactions with iodide ions. This electrostatic complementarity effectively immobilized iodide species at the perovskite interface, mitigating ion migration and strengthening the Sn-I bonds, which enhanced the stability of Sn-Pb perovskites (fig. S3).

To validate the interaction between the designed ligands and SnI_2_, DFT calculations are conducted to investigate the binding energies of different species with SnI_2_/PbI_2_ (Fig. 1D). The results demonstrate that the ligand molecules exhibit higher binding energies with SnI_2_/PbI_2_ compared to FAI, indicating their preferential coordination with SnI_2_/PbI_2_, thereby retarding the crystallization of tin-based perovskites (*58*). Notably, SALC shows the highest binding energy with SnI_2_/PbI_2_, confirming that SALC can form strong interactions with SnI_2_/PbI_2_, consistent with aforementioned conclusions. Meanwhile, DFT results reveal that these ligands exhibit a higher binding energy with SnI_2_ compared to PbI_2_, which favors the regulation of Sn-Pb perovskite crystallization kinetics, ultimately enabling the fabrication of high-quality films. The statistical distribution of PCEs for Sn-Pb PSCs modified with the three ligands is analyzed, and the results align with theoretical predictions. SALC-modified devices demonstrate the highest PCE (fig. S4). In addition, we systematically optimized the additive concentration of the SALC ligand (fig. S5). Henceforth, we focus on investigating the impact of SALC on Sn-Pb perovskites.

When equimolar SALC-SnI_2_ and SALC-PbI_2_ mixtures are separately dissolved in mixed polar solvents (DMF/DMSO), the SALC-SnI_2_ solution remains clear, while the SALC-PbI_2_ solution appears turbid due to partial precipitation of undissolved SALC (Fig. 1E). Furthermore, we also observed that when SALC was dissolved in DMF or DMSO along with SnI_2_ or PbI_2_, the solution state was the same as when it was dissolved in a mixed solvent (fig. S6). Therefore, this difference in solubility behavior further reflects that SALC exhibits preferential coordination with SnI_2_ over PbI_2_, corroborating the strong binding affinity as displayed in the DFT calculations. Fourier transform infrared (FTIR) spectroscopy and proton nuclear magnetic resonance (^1^H NMR) spectroscopy are conducted to investigate the interactions between SALC and SnI_2_/PbI_2_. As shown in Fig. 1F, compared to pure SALC, the N─H stretching vibration, C═O vibration, and C─S vibration peaks in the mixtures of SALC with SnI_2_/PbI_2_ exhibit distinct shifts, indicating interactions mediated by hydrogen bonding and electrostatic attraction. Critically, the peak shifts in the SALC-SnI_2_ mixture (13.0 cm^−1^) are substantially larger than those in the SALC-PbI_2_ mixture (7.3 cm^−1^), demonstrating stronger interactions between SALC and SnI_2_. In the ^1^H NMR spectra (Fig. 1G), the protons associated with the amino group (─NH_2_) of SALC shift to a lower magnetic field upon the addition of SnI_2_/PbI_2_, which can be attributed to the reduction in electron cloud density around the amino group due to the coordination between SALC and SnI_2_/PbI_2_. Notably, the downfield shift of ^1^H peak is more pronounced in the presence of SnI_2_ [0.59 parts per million (ppm)] than with PbI_2_ (0.13 ppm), indicating a stronger coordination interaction between SALC and SnI_2_. To evaluate the antioxidant capacity of the ligand, DFT calculations are used to determine the highest occupied molecular orbital (HOMO) energy levels of SnI_2_ and the SnI_2_-SALC complex in a DMSO solvent (fig. S7). The HOMO energy level of SnI_2_ is calculated to be −5.137 eV, whereas that of the SnI_2_-SALC complexes exhibit a deeper energy level of −5.313 eV. This downward shift in the HOMO energy level indicates that the SnI_2_-SALC complexes are thermodynamically less susceptible to oxidation, demonstrating that the SALC ligands can effectively stabilize SnI_2_ (*59*). The ^119^Sn NMR spectra (fig. S8) show that, compared with the SnI_2_ solution, the addition of SALC induces a notable downfield shift of the Sn^2+^ signal. This result demonstrates that SALC forms a strong coordination with SnI_2_, thereby altering the chemical environment of Sn(II) at the molecular level. We further propose that this distinctive coordination structure may be responsible for the excellent oxidation stability of the system observed in subsequent aging experiments. In addition, we monitored the evolution of the SnI_2_ solution without and with SALC using ultraviolet-visible (UV-Vis) absorption spectroscopy (fig. S9). The oxidation of I^−^ ions to I_3_^−^ is accompanied by the oxidation of Sn^2+^; observing the characteristic peaks of I_3_^−^ allows for judging the oxidation degree of Sn^2+^ (*60*, *61*). After aging for 60 min, the characteristic signals of I_3_^−^ at 293 and 363 nm appeared in the SnI_2_ solution. The SnI_2_ solution with SALC exhibited the I_3_^−^ characteristic peaks only after a longer time interval. This result confirms that SALC can retard the oxidation of Sn^2+^ to a certain extent, which agrees with the theoretical calculations. Based on theoretical calculations and experimental results, the multiple sites of SALC interact with SnI_2_ (fig. S9). The sulfur atom of the thioether group, the oxygen atom of the carboxyl group, and the nitrogen atom of the amino group coordinate with Sn^2+^, while the hydroxyl of the carboxyl group and the ─NH of the amino group form hydrogen bonds with iodide ions. The synergistic interactions from these multiple sites effectively stabilize SnI_2_.

### Modulation of perovskite crystallization growth kinetics

**Fig. 2.**
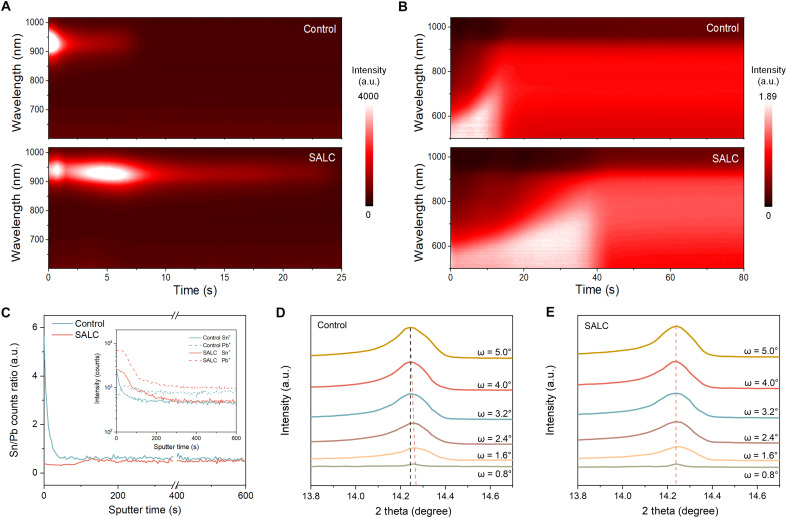
Crystallization process and Sn/Pb distribution. (**A**) In situ PL mapping of the perovskite films without and with SALC during the annealing stage. (**B**) In situ UV-Vis absorption spectra evolution of control and SALC-contained perovskite films during the annealing stage. (**C**) TOF-SIMS depth profiles of Sn-Pb perovskite films without and with SALC, and the corresponding Sn/Pb counts ratio. (**D** and **E**) The GIXRD curves of perovskite films (D) without and (E) with SALC.

To further investigate the influence of SALC ligand on the crystallization kinetics of Sn-Pb perovskites, in situ photoluminescence (PL) spectra during the film annealing stage are recorded (Fig. 2A and fig. S10). For the control film, a broad PL emission signal is observed initially, followed by rapid PL intensity enhancement due to grain growth, which is subsequently quenched within 8 s due to thermally induced defects and nonradiative recombination. In contrast, the SALC-modified films demonstrate narrowed PL emission spectra at the initial stage, showing an increase in initial PL intensity followed by a decrease, subsequently entering a secondary ripening stage where PL emission reintensified. This observation indicates delayed structural reorganization between FA^+^ cations and SnI_2_/PbI_2_, followed by a prolonged quenching process (24 s). In addition, in situ optical absorption spectra are recorded during the annealing procedure (Fig. 2B and fig. S11). Following annealing initiation, the absorption peak intensity increases while the absorption edge redshifts. Notably, the solvent-to-perovskite phase transition time increases from ~15 s in the control film to ~43 s in the SALC-modified film. This delayed transition is attributed to a stabilized intermediate phase formed by SALC coordination with SnI_2_/PbI_2_, which decelerates structural reorganization kinetics. These results confirm that SALC ligands effectively retard the Sn-Pb perovskite crystallization. Typically, during perovskite crystallization, DMSO strongly coordinated with SnI_2_/PbI_2_ to form intermediate phases. Subsequent desorption of DMSO leads to metastable phases that eventually transform into perovskite phases (*16*, *62*). To elucidate the mechanism of the effect of SALC on the crystallization kinetics, we conducted DFT calculations (fig. S12). DFT results reveal stronger binding between SALC and SnI_2_/PbI_2_ compared to DMSO coordination. Therefore, it is expected that these stabilized intermediate phases (SALC-SnI_2_/PbI_2_ and DMSO-SnI_2_/PbI_2_) would facilitate homogeneous nucleation and retarded crystallization kinetics. Notably, the stronger binding affinity of SALC toward SnI_2_ relative to PbI_2_ balances the crystallization rates of Sn and Pb species. The schematic illustration of the crystallization process is provided in fig. S13.

Time-of-flight secondary ion mass spectrometry (TOF-SIMS) is used to probe the depth-dependent compositional distribution within the perovskite films (Fig. 2C). The control films exhibit a Sn-rich surface and a higher Pb content relative to Sn in the bulk, indicating compositional heterogeneity. In contrast, the SALC-modified films demonstrate a homogeneous Sn/Pb ratio within the surface-to-bulk region. This observation aligns well with the proposed mechanism of SALC-mediated crystallization control, which balances the coordination kinetics of Sn^2+^ and Pb^2+^ ions to achieve uniform Sn/Pb distribution throughout the film. Grazing-incidence x-ray diffraction (GIXRD) measurements are performed to in situ detect the compositional gradients of the perovskite films. The GIXRD patterns are recorded at incident angles ranging from 0.8° to 5°, probing compositional variations within the whole perovskite films. As illustrated in Fig. 2 (D and E), for the control sample, the (100) diffraction peak gradually shifts to lower angles with increasing incident angles. This low-angle shift indicates lattice expansion from the surface toward the bulk, consistent with the observed higher Sn/Pb ratio in the bulk. SALC-doped samples display negligible shift in the (100) peak within the penetration depth, demonstrating comparable Sn/Pb ratios at the surface and in the bulk. This confirms a homogeneous distribution of Sn and Pb throughout the perovskite film. To further evaluate the homogeneity of the surface of perovskite films, kelvin probe force microscopy (KPFM) measurements are carried out (fig. S14). As anticipated, the SALC-modified film exhibits a more uniform surface potential distribution compared to the control film. This improvement can be attributed to the SALC-mediated regulation of the perovskite crystallization process, ultimately fostering a homogeneous Sn/Pb distribution.

### Characterization of Sn-Pb perovskite films

**Fig. 3.**
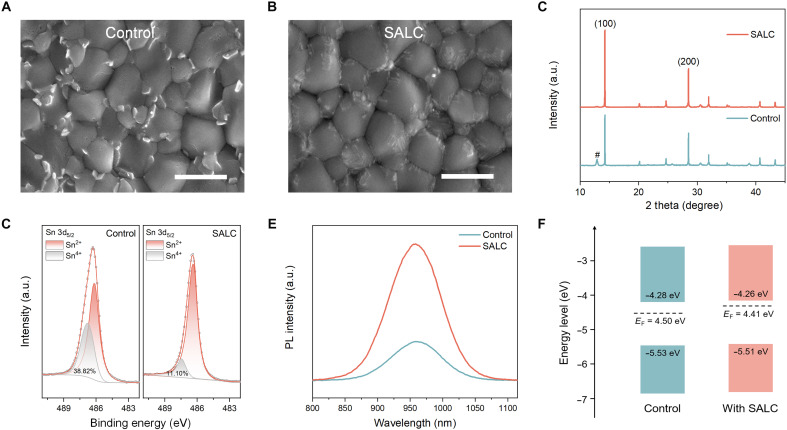
Perovskite film quality and charge carrier dynamics. (**A** and **B**) Top-view SEM images of the perovskite films (A) without and (B) with SALC. Scale bars, 500 nm. (**C**) XRD patterns of the control and SALC-treated perovskite films. The # denotes PbI_2_. (**D**) Sn 3d XPS spectra of control and SALC-modified perovskite films. (**E**) Steady-state PL spectra of the perovskite films without and with SALC. (**F**) Energy-level diagram of the perovskite films without and with SALC.

To further elucidate the impact of SALC on the quality of perovskite films, we systematically examined the structural and morphological characteristics of the films. Scanning electron microscopy (SEM) images (Fig. 3, A and B) demonstrate that the SALC-modified perovskite film exhibited more uniform grains and smoother grain boundaries, compared to the control sample. Furthermore, cross-sectional SEM images reveal that the incorporation of SALC results in the growth of denser and more vertically aligned crystalline domains (fig. S15). X-ray diffraction (XRD) patterns indicate the substantially enhanced crystallinity, with no PbI_2_ peak appeared (Fig. 3C). The correlation between suppressed Sn-rich aggregates and reduced surface Sn signal indicates that SALC mitigates Sn/Pb phase segregation, as evidenced by SEM and TOF-SIMS. The absence of characteristic SnI_2_ peaks supports the interpretation that these features are Sn-rich perovskite domains, whose formation is suppressed by SALC’s coordination-driven crystallization modulation, thereby promoting compositional homogeneity. In addition, atomic force microscopy (AFM) measurements (fig. S16) reveal a notable reduction in surface roughness for the SALC-modified films. The smoother surface is beneficial for interfacial contact, thereby promoting efficient charge transfer and minimizing interfacial recombination losses (*63*). To evaluate the antioxidant properties of the perovskite films, x-ray photoelectron spectroscopy (XPS) analyses were carried out to characterize the Sn^4+^ content in the films. As shown in Fig. 3D, the control film exhibits a higher Sn^4+^ content (gray shaded regions), whereas the SALC-modified films demonstrate a threefold reduction in Sn^4+^ content (from 38.82 to 11.1%). This result confirms that the SALC ligands effectively suppress the oxidation of Sn-Pb perovskite films, consistent with the DFT calculations. In addition, the shifts of the Sn 3d and Pb 4f peaks toward lower binding energies provide further evidence for robust interactions between SALC and the Sn-Pb perovskites (fig. S17). To validate the antioxidant effect of SALC, we monitored the stability of perovskite films exposed to ambient air ( room temperature: 30°C, relative humidity: 40 to 50%). The control film decreases to 63% in absorbance after 30 hours, while the SALC-modified perovskite film exhibits a slight reduction (13%), indicating that SALC effectively enhances the environmental stability of the perovskite films (fig. S18). Consequently, these evidences demonstrate that the primary role of SALC is the coordination-modulated stabilization of Sn^2+^, which decelerates crystallization and yields a high-quality film with superior morphology. The resulting high-quality film, with its reduced density of grain boundaries and defects, intrinsically diminishes the primary sites for Sn^2+^ oxidation, thereby suppressing Sn^4+^ formation.

To investigate the trap-assisted carrier recombination dynamics, steady-state PL spectroscopy measurements was carried out. As shown in Fig. 3E, the SALC-modified perovskite films exhibit a stronger PL intensity compared to the control films, revealing the reduced trap densities and substantially suppressed trap-assisted nonradiative recombination. The Tauc plots derived from absorption spectra confirm an identical optical bandgap of 1.25 eV for both SALC-modified and control perovskite films (fig. S19). In addition, ultraviolet photoelectron spectroscopy (UPS) measurements are used to determine the energy level alignments, including the conduction band (CB), valence band (VB), and Fermi level (*E*_F_) positions (fig. S20). As shown in Fig. 3F, the SALC-modified perovskite film exhibits an upward shift in the *E*_F_ compared to the control one, indicative of more intrinsic characteristics. This shift can be attributed to the improved Sn/Pb homogeneity and reduced defect densities, which collectively optimize the electronic landscape for efficient charge carrier transport.

### Potovoltaic performance of Sn-Pb PSCs

**Fig. 4.**
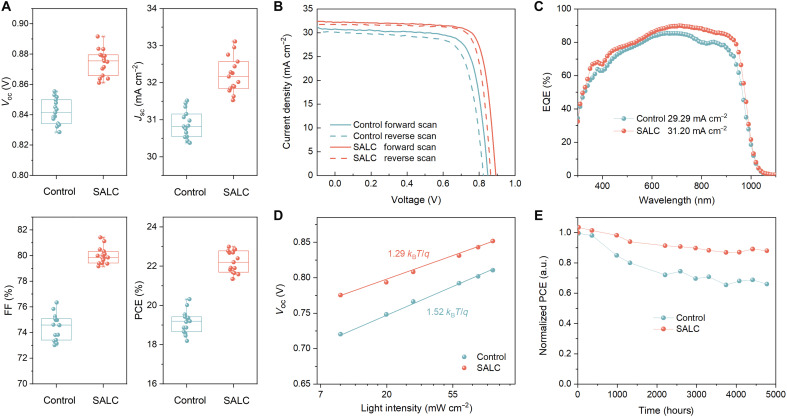
Photovoltaic performance and stability of Sn-Pb PSCs. (**A**) Comparison of the photovoltaic performance between control and SALC-modified devices (15 devices). (**B**) *J*-*V* curves of the best-performing devices without and with SALC. (**C**) EQE spectra of the control and SALC-contained devices. (**D**) The plots of light intensity–dependent *V*_oc_ of Sn-Pb PSCs without and with SALC. (**E**) Long-term stability of unencapsulated Sn-Pb PSCs with and without SALC stored in N_2_ atmosphere.

Based on the above findings, we fabricated Sn-Pb PSCs with the architecture of indium tin oxide (ITO)/poly(3,4-ethylenedioxythiophene):poly(styrene-sulfonate) (PEDOT:PSS)/Sn-Pb perovskite (Cs_0.1_FA_0.6_MA_0.3_Pb_0.5_Sn_0.5_I_3_)/fullerene-C_60_ (C_60_)/bathocuproine (BCP)/copper (Cu) to further evaluate the impact of SALC ligands on the photovoltaic device performance. Statistical analyses of the photovoltaic parameters for devices with and without SALC are summarized in Fig. 4A. Compared to the control devices, SALC-treated devices exhibit superior reproducibility and enhanced performance, particularly in fill factor (FF) and *V*_OC_. The current density–voltage (*J*-*V*) curves of the champion devices are shown in Fig. 4B. The control devices achieve a PCE of 19.62% with a short-circuit current density (*J*_SC_) of 30.75 mA cm^−2^, a *V*_OC_ of 0.847 V, and an FF of 75.20%, but the SALC-modified champion device delivered a PCE of 22.99%, with a *V*_OC_ of 0.892 V, a *J*_SC_ of 32.57 mA cm^−2^, and an FF of 79.16%. The increase in *J*_SC_ of SALC devices aligned with the enhanced photoresponse in the 700 to 1000 nm region of the external quantum efficiency (EQE) spectra (Fig. 4C), attributing to improved photon harvesting derived from high-quality perovskite films. To gain more insights into the impact of SALC ligands on the improvements in *V*_OC_ and FF, we investigated the charge transport dynamics of the devices by measuring the *J*-*V* characteristics of Sn-Pb PSCs under varying light intensities. The dependences of *V*_OC_ on light intensities are plotted in Fig. 4D. The ideality factor (*n*), which reflects the carrier recombination mechanism within the device, is determined to be 1.29 for SALC-modified devices, considerably lower than the 1.52 observed for control devices. This reduction emphasizes a substantial suppression of trap-assisted Shockley-Read-Hall (SRH) recombination, attributing to the decreased defect densities and matched energy level alignment.

To quantitatively characterize the trap state densities of perovskite films, we constructed hole-only and electron-only devices and used the space-charge-limited current (SCLC) model (fig. S21). For the control films, the trap densities (*N*_trap_) derived from hole-only and electron-only devices were 8.13 × 10^15^ and 6.52 × 10^15^ cm^−3^, respectively. In contrast, the SALC-modified films exhibit considerably reduced trap densities of 4.04 × 10^15^ (hole-only) and 3.16 × 10^15^ cm^−3^ (electron-only). Furthermore, we conducted the thermal admittance spectroscopy measurements to characterize the trap density of states; the shower trap states (0.4 to 0.5 eV) and deep-level defect densities (0.5 to 0.55 eV) were substantially reduced in the SALC-modified devices (fig. S22). These results consolidate that SALC effectively suppresses defect formation and improves the optoelectronic quality of the perovskite films.

Mott-Schottky analysis manifest that the SALC-modified Sn-Pb perovskite devices yield a higher built-in potential (*V*_bi_ = 0.753 V) compared to the control device (*V*_bi_ = 0.696 V) (fig. S23). This enhanced built-in potential facilitates carrier extraction and reduces nonradiative recombination losses, which correlated with the upward-shifted Fermi level and reduced defect state densities. Moreover, we analyzed the diode characteristics by measuring the *J*-*V* curves under dark conditions (fig. S24). The SALC-modified device demonstrates a dramatically reduced reverse saturation current density relative to the control, confirming suppressed leakage current, improved current injection efficiency, and inhibited nonradiative recombination——all of which synergistically enhance charge transfer dynamics. In transient photocurrent (TPC) measurements (fig. S25), SALC-modified devices displayed a reduced decay lifetime (from 850 to 470 ns), suggesting that SALC modification effectively suppresses nonradiative recombination at interfaces, lowers defect state densities, and promotes charge extraction. These improvements collectively contribute to the observed enhancements in *V*_OC_ and FF. To evaluate the long-term stability of the unencapsulated Sn-Pb PSCs with or without SALC, we systematically monitored the PCE evolution under a N_2_ atmosphere at ambient temperature (Fig. 4E). The SALC-modified devices demonstrate exceptional durability, retaining 88% of the initial PCE after 4776 hours, whereas the control devices degrade to 66% under the same conditions. The stability improvement can be attributed to the high-quality films with homogeneous Sn/Pb distribution and suppressed Sn^4+^-related defects.

### Performance and stability of all-perovskite TSCs

**Fig. 5.**
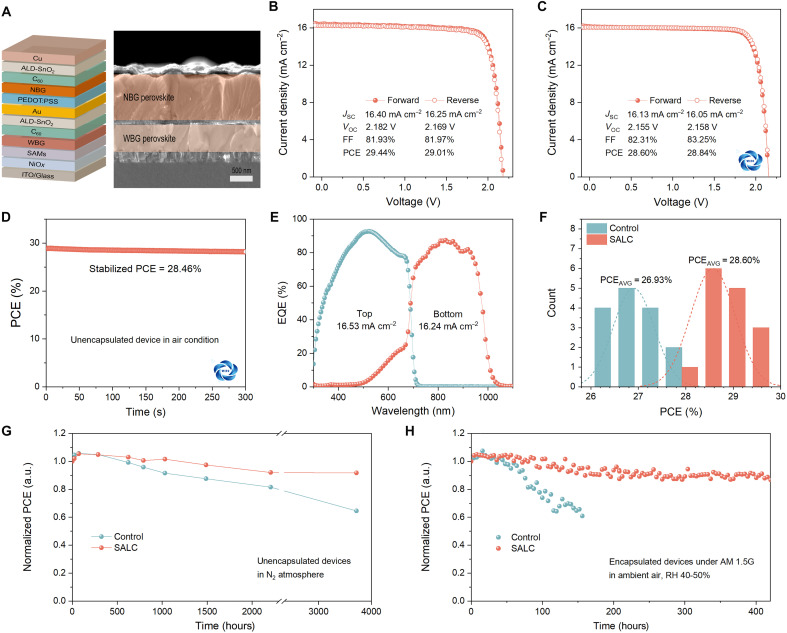
Photovoltaic performance and stability of all-perovskite TSCs. (**A**) Device architecture diagram and corresponding cross-section SEM image of the all-perovskite TSC. (**B**) *J*-*V* curves of the champion TSC under forward and reverse voltage scans. (**C**) The certified *J*-*V* curves of SALC-modified tandem device. (**D**) The certified MPP tracking of unencapsulated SALC-treated TSC in the air condition. (**E**) EQE spectra of the champion tandem device. (**F**) The PCE distribution statistics of control and SALC-contained tandem devices. (**G**) Long-term stability of the unencapsulated tandem devices without and with SALC. (**H**) MPP tracking stability of the tandem devices without and with SALC under 1 sun illumination in ambient air. RH, relative humidity.

Motivated by the performance enhancement of SALC-modified Sn-Pb PSCs, we integrated NBG subcells with 1.77-eV Cs_0.2_FA_0.8_PbI_1.8_Br_1.2_ WBG subcells to fabricate two-terminal all-perovskite TSCs, with the device architecture of ITO/NiO*_x_*/[4-(3,6-dimethyl-9*H*-carbazol-9-yl)butyl]phosphonic acid (Me-4PACz):[2-(3,6-dimethoxy-*9*H-carbazol-9-yl)ethyl]phosphonic acid (MeO-2PACz)/WBG perovskite/C_60_/SnO_2_/Au/PEDOT:PSS/NBG perovskite/C_60_/SnO_2_/Cu (Fig. 5A). The cross-sectional SEM image of the corresponding tandem device is displayed in Fig. 5A. The thicknesses of WBG and NBG perovskites are ~450 and 800 nm. The single-junction WBG PSCs achieved a PCE of 19.60% with a *V*_OC_ of 1.359 V (fig. S26). As shown in Fig. 5B, the champion SALC-incorporated tandem cell delivered an impressive PCE of 29.44% (29.01%), with a *V*_OC_ of 2.182 (2.169) V, a *J*_SC_ of 16.40 (16.25) mA cm^−2^, and an FF of 81.93% (81.97%). One best-performing SALC tandem device without encapsulation achieves a certified PCE of 28.84% (28.60%) with a stabilized PCE of 28.46% (Fig. 5, C and D, and fig. S27). The EQE spectra of the WBG and NBG subcells in the tandem device yielded integrated *J*_SC_ values of 16.53 and 16.24 mA cm^−2^, respectively, demonstrating well-matched photocurrent generation (Fig. 5E). Statistical analyses of multiple devices show that SALC-modified tandem cells yield an average PCE of 28.60%, substantially outperforming the control tandems (26.93%), along with superior reproducibility (Fig. 5F). To assess long-term shelf stability, we monitored the PCE evolution of unencapsulated tandem devices stored in a nitrogen atmosphere at ambient temperature. As illustrated in Fig. 5G, the control tandem device retains only 77% of the initial PCE after 3720 hours, whereas the SALC-modified tandem device maintains 92% of the original PCE, highlighting the strengthened durability. Meanwhile, the encapsulated tandem devices exposed to ambient air under 1 sun illumination are tracked to evaluated the operational stability. After 156 hours of MPPT tracking, the control device degrades to 60.93% of the initial PCE, while the SALC-contained device retains 86.67% of the original PCE after 420 hours, suggesting dramatically enhanced operational stability (Fig. 5H). These results declare that homogeneous Sn/Pb distribution in the perovskite absorber is pivotal to achieving both high efficiency and robust stability in all-perovskite tandem devices. Further pushing the stability ceiling toward the highest benchmarks will require addressing several interconnected challenges: optimizing the stability of charge transport layers and interconnection interfaces, and implementing advanced encapsulation schemes to combat extrinsic degradation.

## DISCUSSION

In summary, this work highlights the effectiveness of a multifunctional Sn-coordinating ligand to modulate the crystallization kinetics of Sn-Pb perovskites, achieving high-quality perovskite films with homogeneous Sn/Pb distribution, decreased defect density, and suppressed Sn^2+^ oxidation. Furthermore, the SALC could optimize the band structure, mitigate nonradiative recombination and minimize energy loss. Consequently, the SALC-modified Sn-Pb PSC delivered high efficiency of 22.99% with only 0.358 V of *V*_OC_ loss. The resulting champion all-perovskite tandem device achieved a remarkable PCE of 29.44% (certified 28.84% without encapsulation), with markedly strengthened shelf stability and threefold improvement in long-term operational stability. Our work offers valuable insights into ligand design for advancing Sn-Pb perovskites and tandem optoelectronics.

## MATERIALS AND METHODS

### Materials

PEDOT: PSS (CLEVIOS P VP AI 4083) was obtained from Heraeus. DMF (anhydrous, 99.8%), DMSO (anhydrous, 99.9%), isopropanol (IPA; anhydrous, 99.5%), ethyl alcohol (EtOH; anhydrous, 99.5%), Sn powder, SnF_2_, ammonium thiocyanate (NH_4_SCN), cesium iodide (CsI), and LC were purchased from Sigma-Aldrich. FAI and methylammonium iodide (MAI) were purchased from Greatcell. Lead iodide (PbI_2_), lead bromide (PbBr_2_), Me-4PACz, MeO-2PACz, SALC, and SBLC were purchased from TCI. Propane-1,3-diammonium iodide (PDAI_2_), ethylenediamine dihydroiodide (EDAI_2_), C_60_, and BCP were purchased from Xi’an Polymer Light Technology Corp. Unless otherwise stated, all materials were used as received without further purification. Anisole was purchased from J&K.

### Precursor preparation

For the NBG perovskite (Cs_0.1_FA_0.6_MA_0.3_Pb_0.5_Sn_0.5_I_3_), 1.8 M precursor was prepared by mixing 0.180 mmol CsI, 1.08 mmol FAI, 0.540 mmol MAI, 0.900 mmol PbI_2_, 0.900 mmol SnI_2_, 0.090 mmol SnF_2_, and 0.036 mmol NH_4_SCN in 1 ml of DMF:DMSO (3:1, v/v). For the target precursor, 0.4 mol % of SALC was added to the precursor. The precursors were stirred overnight and filtered with a 0.22-μm polytetrafluoroethylene filter before using.

For the WBG perovskite [FA_0.8_Cs_0.2_Pb(I_0.6_Br_0.4_)_3_], 1.2 M precursor was prepared by dissolving 0.960 mmol FAI, 0.240 mmol CsI, 0.480 mmol PbBr_2_, and 0.720 mmol PbI_2_ in 1 ml of mixed solvent with DMF:DMSO (3:1, v/v) and stirred overnight.

### Device fabrication

#### Single-junction NBG PSC fabrication

The ITO-coated glasses were cleaned with deionized water, IPA, and EtOH by an ultrasonic cleaner successively. The dried ITO glasses were treated by UV ozone cleaner for 15 min. PEDOT:PSS dispersion was filtered through a 0.45-μm polyvinylidene difluoride filter and then spin-coated on the substrates at 4000 rpm for 30 s, followed by annealing at 150°C for 15 min in air. After that, the substrates were transferred into a nitrogen-protected glovebox, the prepared perovskite precursors were spun onto the substrates in a two-step process at 2000 rpm for 10 s and 4000 rpm for 40 s, and 300 μl of anisole was dropped at 15 s before the end of procedure. Then, the wet films were baked at 100°C for 10 min. After cooling, the EDAI_2_ solution was spin-coated on the perovskite film at 4000 rpm for 30 s, followed by heating at 100°C for 5 min. After completing the above preparation process, the substrates were transferred to the vacuum chamber. C_60_ (20 nm), BCP (7 nm), and Cu (100 nm) electrode were subsequently deposited by thermal evaporation. The active area of the devices is 0.08 mm^2^.

#### All-perovskite TSC fabrication

The cleaned and dried ITO substrates were treated by UV-ozone plasma for 15 min. The NiO*_x_* aqueous solution was spin-coated onto the substrates at 5000 rpm for 20 s and then dried at 150°C for 30 min in air. The following steps are performed in the glovebox. The mixed Me-4PACz:MeO-2PACz (1:1, v/v) ethanol solution (0.5 mg/ml) was spin-coated on the NiO*_x_* layer at 3000 rpm for 30 s and then annealed at 100°C for 10 min. The perovskite films were prepared with a two-step spinning procedure (1000 rpm for 10 s and 5000 rpm for 40 s). Anisole was dropped on the wet film at 20 s of the second step. The as-prepared perovskite films were placed on a hotplate at 100°C for 10 min. After cooling down to the room temperature, the substrates were posttreated by spinning PDAI_2_ solution at 4000 rpm for 20 s and then annealed at 100°C for 5 min. Then, the substrates were transferred to the vacuum chamber, C_60_ (20 nm) was deposited by thermal evaporation. Subsequently, SnO_2_ (20 nm) was fabricated by atomic layer deposition. Next, the Au (1 nm) was thermally evaporated on the substrates. After that, PEDOT:PSS (diluted with IPA) solution was spin-coated on the WBG subcells at 4000 rpm for 30 s and then annealed at 110°C for 10 min in air. Next, the cooled WBG subcells were transferred to N_2_-filled glovebox to fabricate NBG subcells. The NBG subcells were prepared as in the abovementioned steps.

#### Characterizations

^1^H NMR spectra were measured with a 400-MHz Bruker Magnetic Resonance spectrometer. XPS and UPS measurements were conducted using an ESCALab250Xi multifunctional photoelectron spectrometer (Thermo Fisher Scientific) using a He Iα source (*h*ν = 21.22 eV). FTIR spectra were obtained from a Bruker Invenio R spectrometer. In situ PL measurements were performed in a N_2_-filled glovebox with a laser (405 nm) as the light source and a fiber-coupled spectrometer as the recorder (Ocean Optics). TOF-SIMS experiments were conducted on an IONTOF M6 instrument with dual-beam profiling (1 keV Cs^+^ and 30 keV Bi^3+^). The sputtering was conducted by Cs on an area of 180 μm by 180 μm, while the analysis area was 100 μm by 100 μm. The top-view and cross-section SEM images were measured by a field emission SEM instrument (Thermo Fisher Scientific Apero 2). GIXRD and XRD patterns were conducted by a Bruker D8 Advance diffractometer, equipped with Cu Kα radiation (wavelength of 1.54 Å). The steady-state PL was obtained from an FLS 980 fluorescence spectrometer (Edinburgh), with a 532-nm pulse laser. AFM and KPFM measurements were conducted on a Bruker Dimension lcon AFM. ^119^Sn NMR spectra were recorded on a VNMRS 600-MHz spectrometer (Varian). UV-Vis absorption spectra were measured using a UV-Vis spectrophotometer (UV2600i, Shimadzu, Japan). The in situ UV-Vis absorption spectra were measured via a home-built spectrometer system equipped with a focused light from halogen lamp and optical fiber connected with a spectrometer. Mott-Schottky and TPC measurements were performed using a multifunctional photoelectric characterization suit (FLUXiM Paios). The *J*-*V* characteristics were measured using a Keithley 2400 source meter under AM 1.5G illumination with a solar simulator (Enli Tech). The trap-filled limit voltage (*V*_TFL_) was determined from the intersection of the linear and nonlinear regions in the current-voltage (*I*-*V*) curves of SCLC measurements. The trap state densities (*N*_trap_) were calculated using the equation: *N*_trap_ = 2εε_0_*V*_TFL_/*qL*^2^, where ε is the relative permittivity, *L* is the film thickness, and *q* is the elementary charge. The EQE spectra were measured with a QE-R system (Enli Tech), with monochromatic light focused on the device pixel and a chopper frequency of 20 Hz. The shelf-stability data of Sn-Pb PSCs and all-perovskite TSCs for each condition are derived from representative devices. Operational stability tests were carried out under AM 1.5G illumination (100 mW cm^−2^, without UV filter) using an MPPT instrument (Wuhan Jiuyao) in ambient air.

#### DFT simulations

DFT, as implemented in Vienna ab initio simulation package, was used to carry out the calculations presented here. The projector augmented wave method was used to treat the effective interaction of the core electrons and nucleus with the valence electrons, while exchange and correlation were described using the Perdew-Burke-Ernzerhof functional. The cutoff energy is set at 600 eV for the plane-wave basis restriction in all calculations. *K*-points are sampled under the Monkhorst-Pack scheme for the Brillouin-zone integration (*K*-points were sampled using the gamma point). In all calculations, the forces acting on all atoms are <0.02 eV Å^−1^ in fully relaxed structures, and self-consistency accuracy of 10^−5^ eV is reached for electronic loops. The HOMO energy levels were obtained using Gaussian 16 with the B3LYP functional. The 6-31G(d,p) basis set was used for C, H, N, O, and S atoms, while LANL2DZ was used for Sn and I atoms. While these absolute energies are method-dependent, the consistent computational approach ensures the reliability of relative comparisons between systems. The observed energy shifts provide valuable insights into electronic structure modifications upon coordination.

The binding energy (*E*_b_) was calculated as follows

$$E_{b}=E_{\text{tot}}-E_{A}-E_{B}$$

where *E*_tot_ is the total energy of the system, and *E*_A_ and *E*_B_ represent the total energy of free species, respectively.

## Supplementary Materials

### This PDF file includes:

Figs. S1 to S27 Table S1 References
