# Supplementary material for: Enhancing Sn-Pb perovskite homogeneity via thioether coordination for efficient and stable all-perovskite tandem solar cells
Source: Sci Adv. 2026 Jul 10;12(28):eaeb8790. doi: 10.1126/sciadv.aeb8790 (PMC13353367; doi:10.1126/sciadv.aeb8790)
Supplement: Supplementary file 1 — Figs. S1 to S27 Table S1 References [file sciadv.aeb8790_sm.pdf]

Science Advances
AAAS

Supplementary Materials for

Enhancing Sn-Pb perovskite homogeneity via thioether coordination for efficient and stable all-perovskite tandem solar cells

Lijuan He *et al.*

Corresponding author: Wei Zhang, wz0003@surrey.ac.uk; Yang Bai, y.bai@siat.ac.cn;
Hui-Ming Cheng, hm.cheng@siat.ac.cn

Sci. Adv. **12**, eaeb8790 (2026)
DOI: 10.1126/sciadv.aeb8790

This PDF file includes:

Figs. S1 to S27
Table S1
References

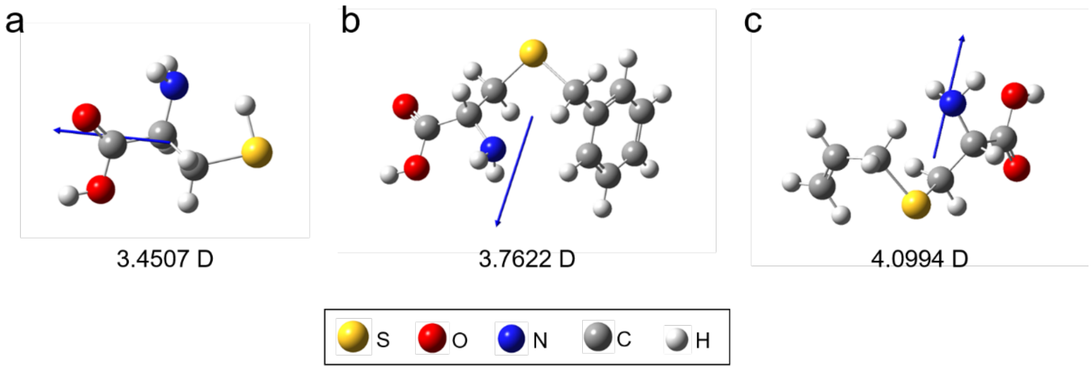

Fig. S1. Molecular dipole moments. The dipole moments of (a) LC (b) SBLC and (c) SALC molecules.

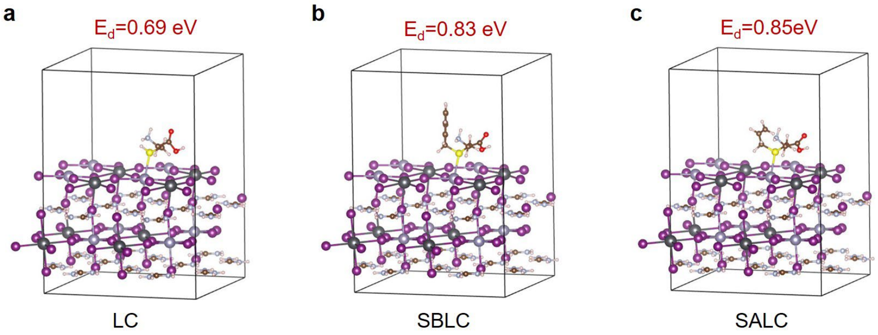

Fig. S2. The adsorption energy of molecules on the surface of perovskite. The adsorption energies (E_{ad}) of the (a) LC (b) SBLC and (c) SALC absorbed on the perovskite surfaces.

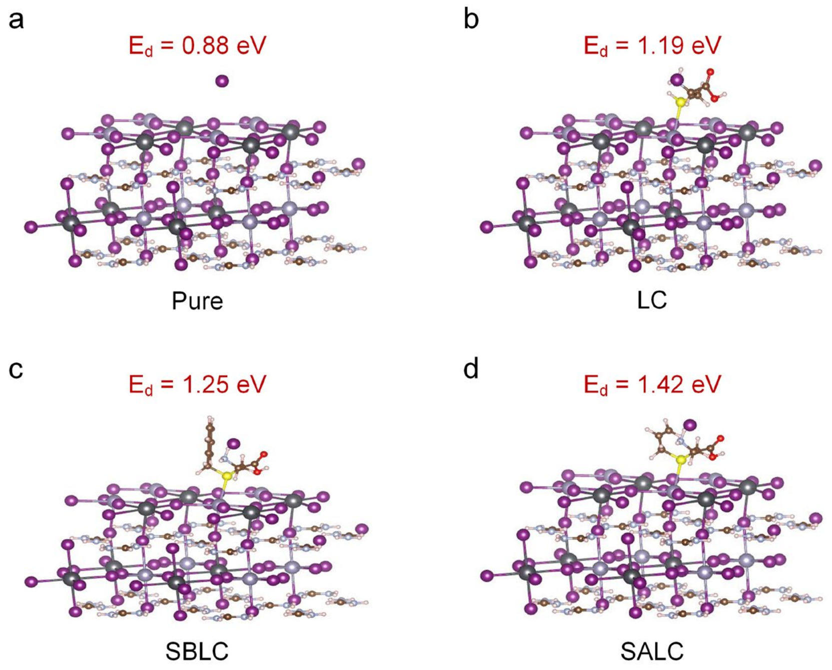

Fig. S3. Prediction models for iodide ion migration. DFT-predicted configurations of iodine migration on the (a) control (b) LC (c) SBLC and (d) SALC occupied perovskite surfaces.

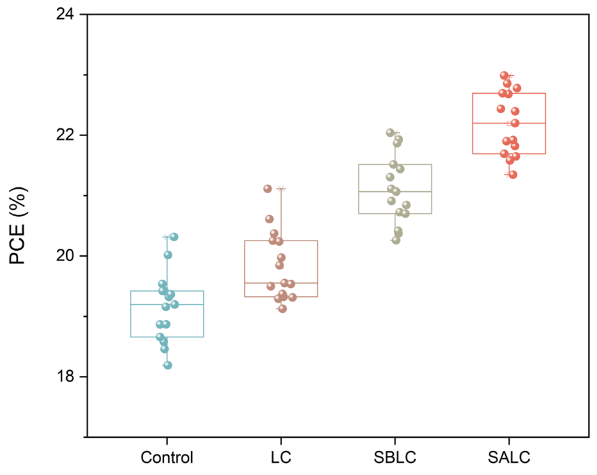

Fig. S4. Performance statistics of different devices. Performance statistics of the control, LC, SBLC and SALC-treated Sn-Pb PSCs (Device architecture of ITO/ poly(3,4-ethylenedioxythiophene):poly(styrene-sulfonate) (PEDOT:PSS) /Sn-Pb perovskite ($Cs_{0.1}FA_{0.6}MA_{0.3}Pb_{0.5}Sn_{0.5}I_3$) / fullerene-$C_{60}$ (C_{60}) /bathocuproine (BCP) /copper (Cu)).

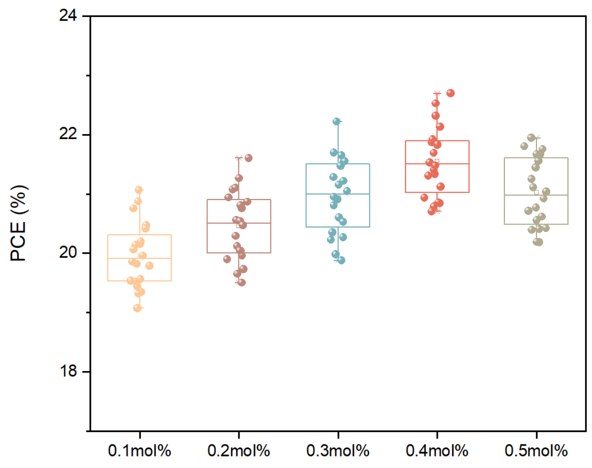

Fig. S5. Performance statistics of devices with different doping ratios. Performance statistics of the Sn-Pb PSCs processing with various SALC doping concentrations (molar ratio vs SnI_2, dissolved in DMF:DMSO 3:1).

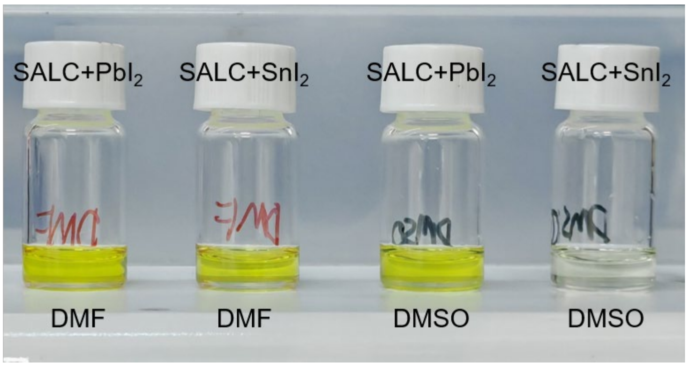

Fig. S6. Photographs of the solutions. SALC was mixed separately with PbI_2 and SnI_2, each dissolved in DMF or DMSO solvent.

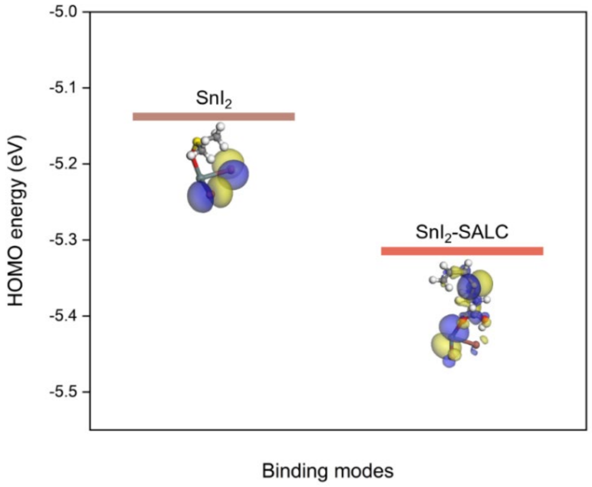

Fig. S7. The HOMO energy level of the molecules. The calculated HOMO level of the SnI_2 and SnI_2-SALC complexes in the DMSO solvent.

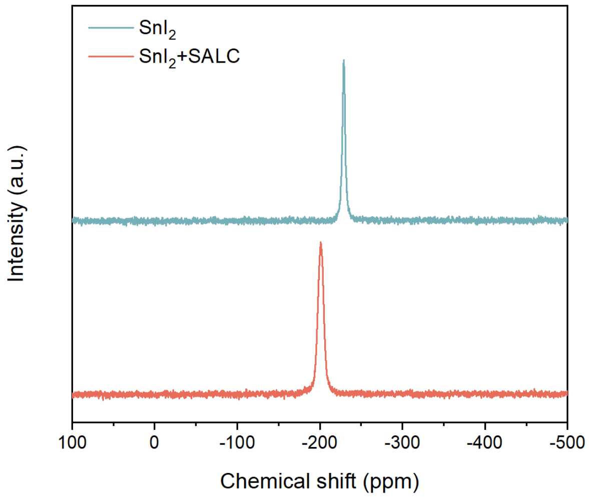

Fig. S8. Chemical interaction of SALC with SnI_2. ^{119}Sn NMR spectra of SnI_2 and SnI_2 with SALC dissolved in a solvent mixture of DMSO-d_6 and DMF-d_7 (1:3, v:v).

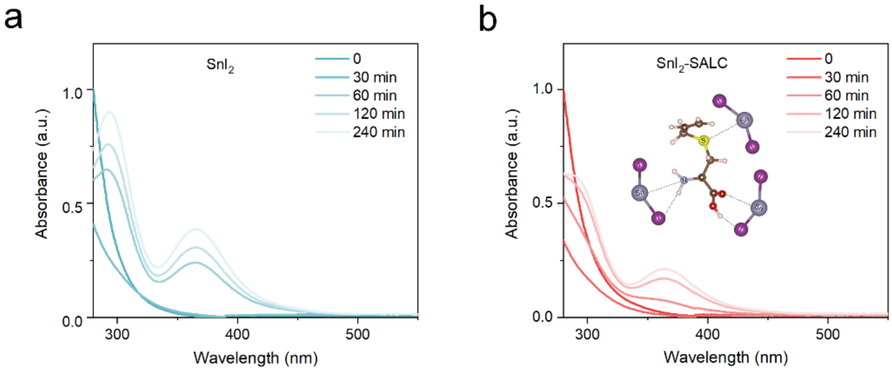

Fig. S9. Evolution of absorption intensity of solution. (a) SnI_2 in mixed solution, (b) SnI_2 with SALC in mixed solution. The insert of (b) shows the coordination structure between SnI_2 and SALC.

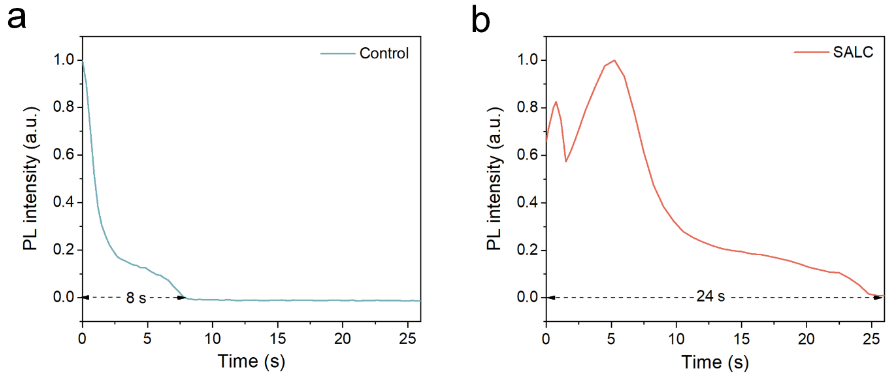

Fig. S10. Evolution of the PL intensity of perovskite films. The corresponding devotions of PL intensity for the control and SALC-containing perovskite films.

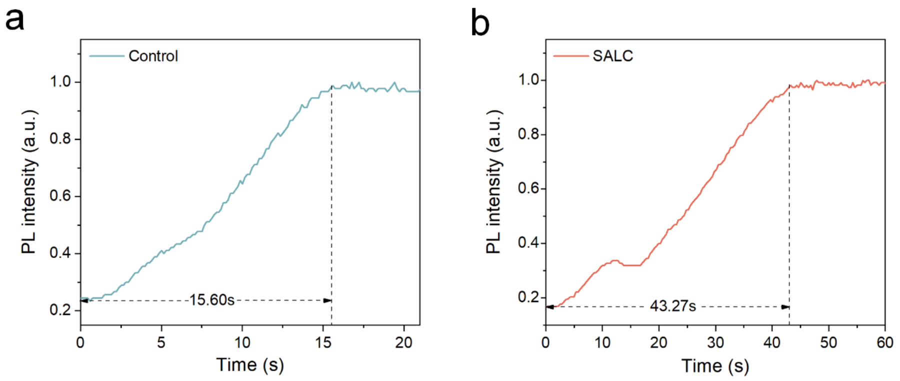

Fig. S11. Evolution of the absorption intensity of perovskite films. The corresponding devotions of absorption for the control and SALC-containing perovskite films.

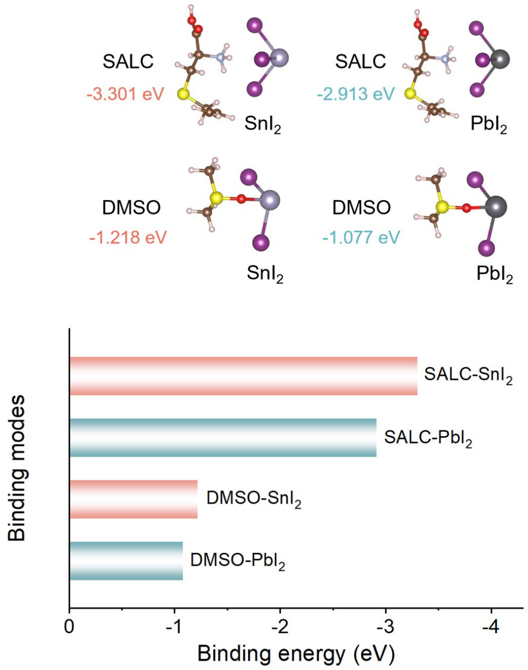

Fig. S12. The binding energies of different molecular complexes. The binding energies of DMSO-PbI_2, DMSO-SnI_2, SALC- PbI_2 and SALC- SnI_2 complexes.

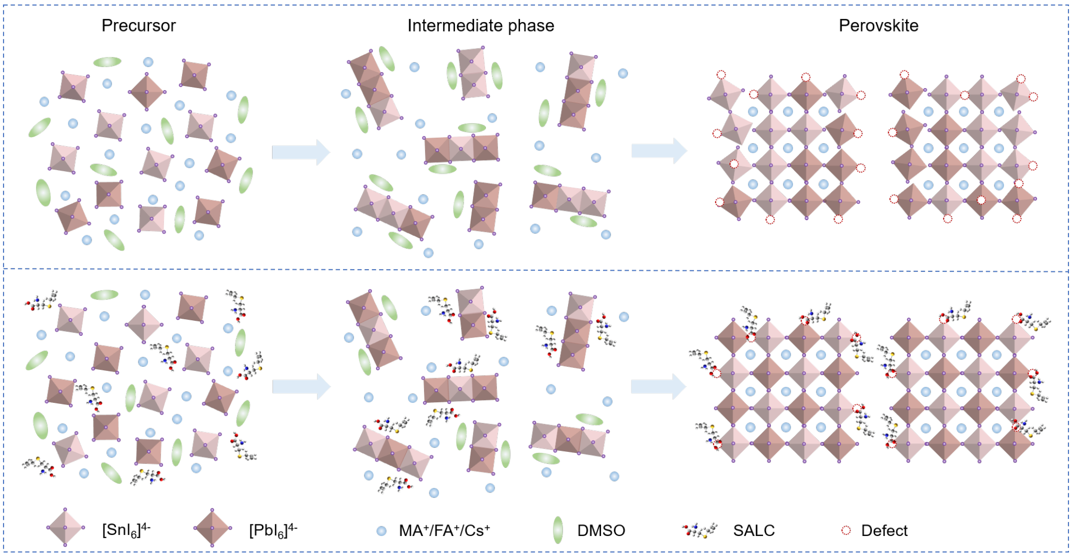

Fig. S13. Schematic diagrams of crystallization kinetics. Schematic diagrams of the nucleation and crystallization dynamics in Sn-Pb perovskites, correlating with Sn/Pb spatial distribution.

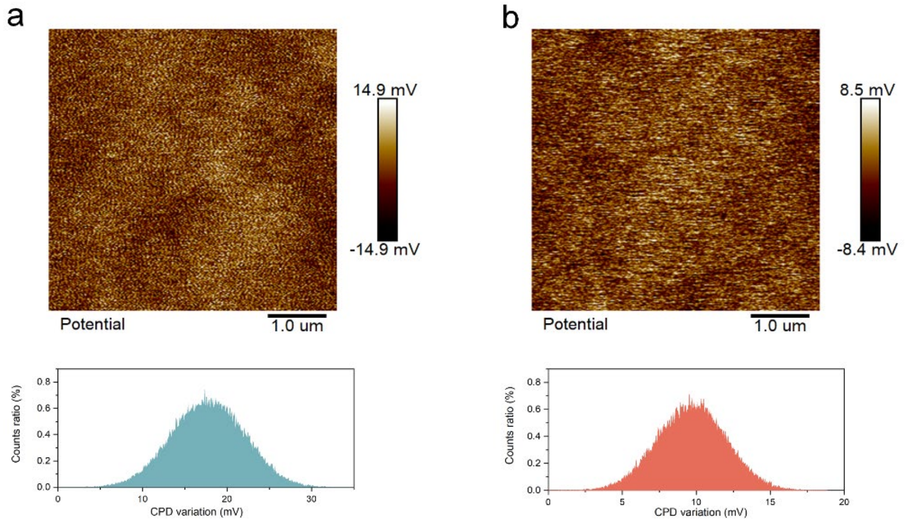

Fig. S14. KPFM image of perovskite films. Perovskite films (a) without and (b) with SALC. The potential distribution curves of the corresponding perovskite films are shown below the images.

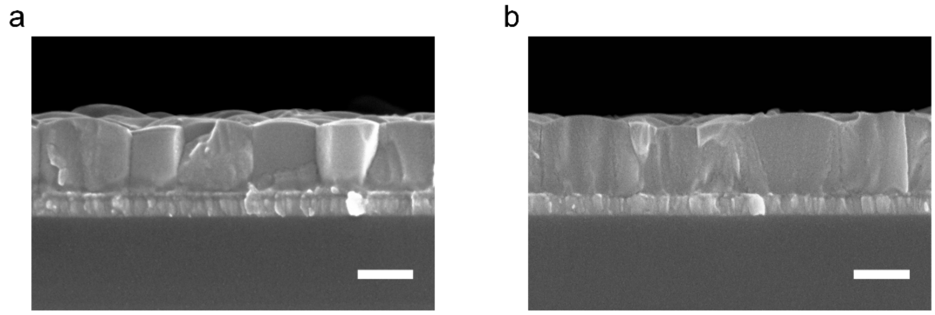

Fig. S15. Cross-sectional SEM images of perovskite films fabricated on PEDOT:PSS-coated ITO substrates. Perovskite film (a) without and (b) with SALC. The scale bar is 500 nm.

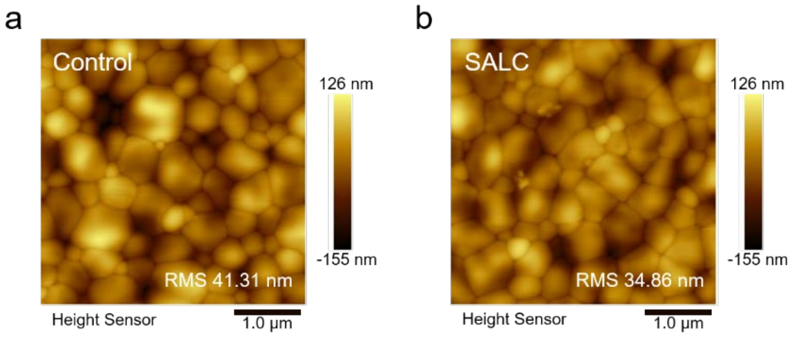

Fig. S16. AFM images of the perovskite films. (a) control and (b) SALC-doped modified perovskite films.

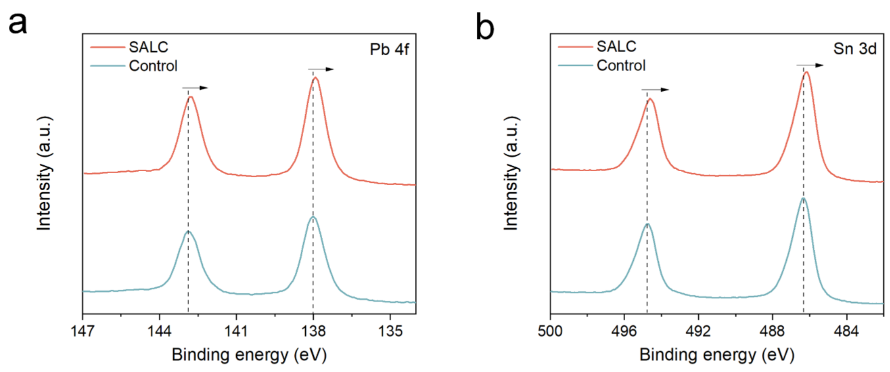

Fig. S17. XPS spectra of the perovskite films. (a) Pb 4f and (b) Sn 3d core-level of the control and with SALC-modified films.

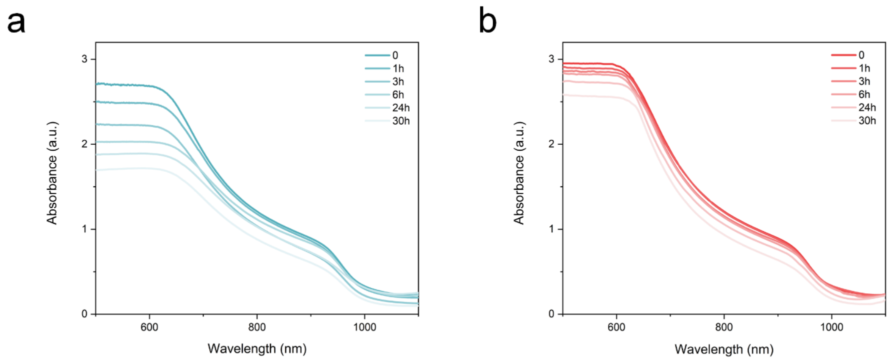

Fig. S18. UV absorption spectra evolution of perovskite films. a) control film and b) SALC-modified film with $EDAI_2$ post-treatment exposed to ambient air (RT: 30℃, RH: 40%-50%).

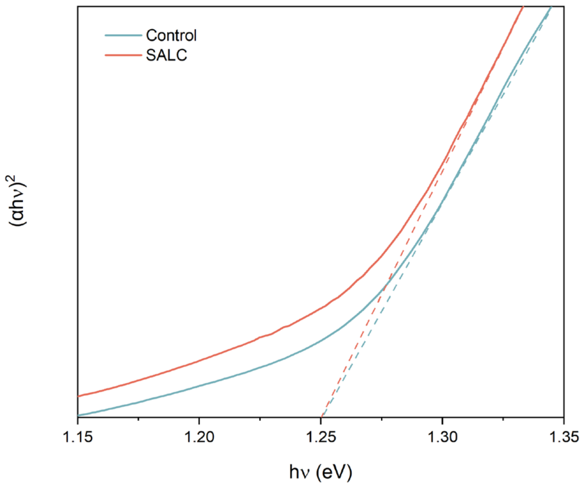

Fig. S19. Tauc plots of perovskite films. Perovskite films ($Cs_{0.1}FA_{0.6}MA_{0.3}Pb_{0.5}Sn_{0.5}I_3$) with and without SALC. The optical bandgaps were calculated to be both 1.25 eV.

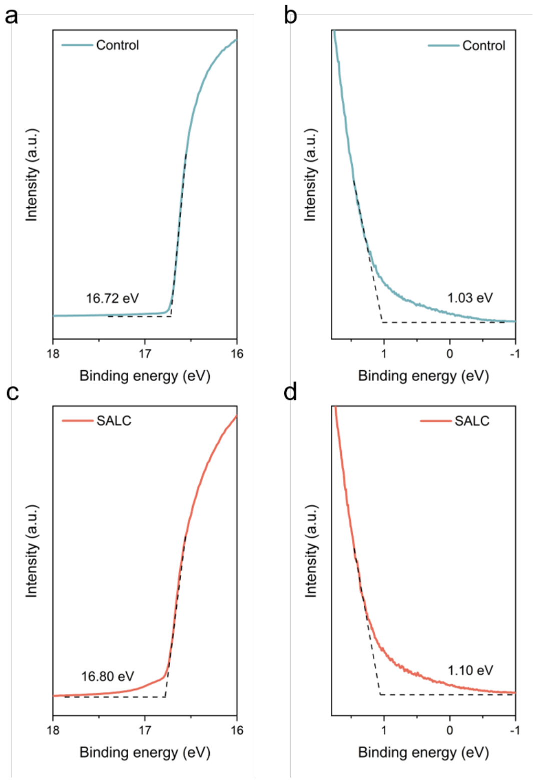

Fig. S20. UPS spectra of perovskite film. (a-b) Control perovskite film, (c-d) Perovskite film with SALC.

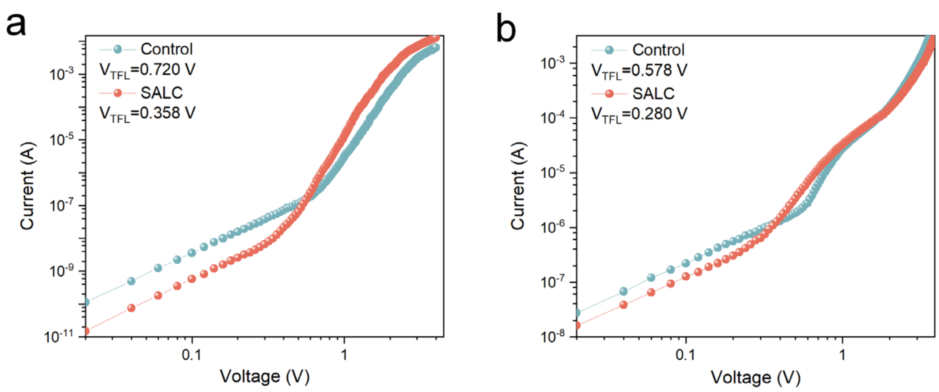

Fig. S21. *J-V* curves of different devices. (a) hole-only devices with and without SALC and (b) electron-only devices with and without SALC.

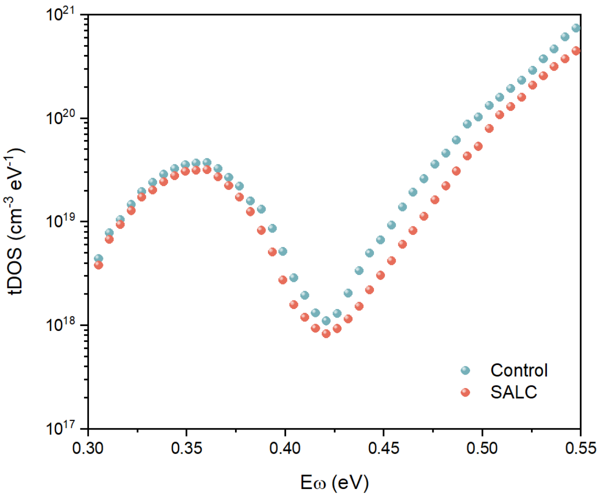

Fig. S22. tDOS plots of different devices. tDOS plots of control and SALC-modified Sn-Pb PSCs.

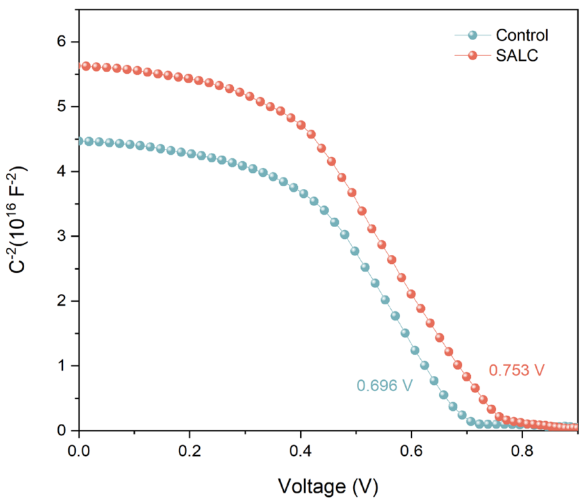

Fig. S23. Mott-Schottky plots of different devices. Mott-Schottky plots of Sn-Pb PSCs with and without SALC.

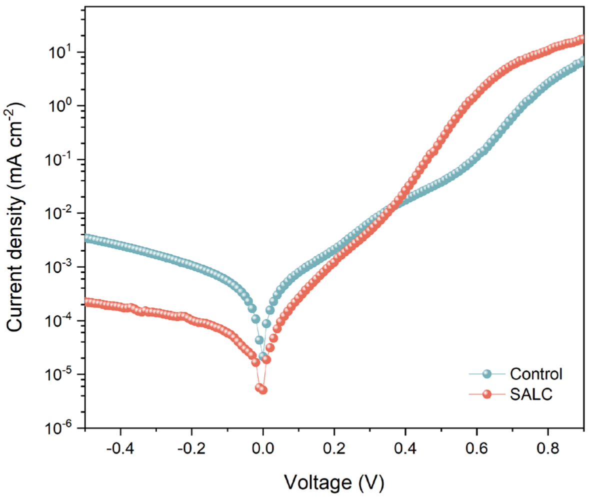

Fig. S24. Dark *J-V* characteristics of different devices. Mott-Schottky plots of the control and SALC-modified devices.

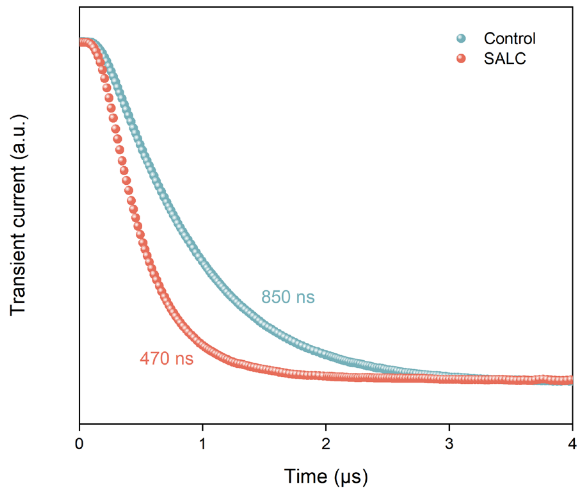

Fig. S25. Transient photocurrent curves of different devices. Transient photocurrent curves of the control and SALC-treated devices.

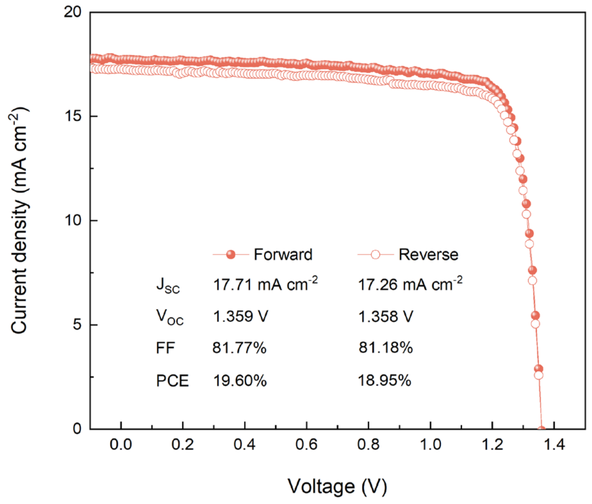

Fig. S26. *J-V* characteristics of WBG PSC. *J-V* curves of single-junction 1.77eV-WBG perovskite solar cell.

国家光伏质检中心

检验检测报告

TEST REPORT

No:2025DMCS20094

产品名称<br>SAMPLE	全钙钛矿叠层太阳能电池<br>All-perovskite Tandem Solar Cells
规格型号<br>MODEL/TYPE	15 mm *15 mm
委托单位<br>APPLICANT	中国科学院深圳先进技术研究院 / 深圳理工大学<br>Shenzhen Institute of Advanced Technology, Chinese Academy of Sciences / Shenzhen University of Advanced Technology

无锡市检验检测认证研究院

Wuxi Institute of Inspection, Testing and Certification

国家太阳能光伏产品质量检验检测中心

National Center of Inspection on Solar Photovoltaic Products Quality

检 验 检 测 结 果

Test Results

序号<br>Clause	检验项目<br>Test item(s)	单位<br>Unit	技术要求<br>Technical requirements	结果<br>Results	单项评价<br>Verdict<br>Pass/Fail
1	电流-电压特性的测量(正扫)<br>Current-voltage characteristic measurement (Forward scan)	—	在标准试验条件下（样品温度：25℃±2℃，辐照度：1000W/ m²，标准太阳光谱辐照分布符合 IEC 60904-3 规定），测量样品随负荷变化的电流-电压特性。<br>At STC (module temperature: 25℃±2℃, irradiance: 1000W/m², standard solar spectral irradiance distribution corresponds to IEC60904-3), measure the current-voltage characteristics of the cell with the variation of load.	—	—
1.1	开路电压 Voc<br>Open-circuit voltage, Voc	V	—	2.155	—
1.2	短路电流 Isc<br>Short-circuit current, Isc	mA	—	0.8158	—
1.3	最大功率 Pmax<br>Maximum-power, Pmax	mW	—	1.447	—
1.4	最大功率点电压 Vmp<br>Maximum-power voltage, Vmp	V	—	1.860	—
1.5	最大功率点电流 Imp<br>Maximum-power current, Imp	mA	—	0.7777	—
1.6	填充因子 FF，%<br>Fill factor FF, %	—	—	82.31	—
1.7	转换效率 η，%<br>Conversion efficiency η, %	—	$\eta = \frac{P_{max}}{1000W/m^2 \times S} \times 100\%$<br>S 为掩膜板面积/Area S is determined by mask	28.60	—

备注：正扫扫描方向为-0.1V~2.24V，步进 0.02V，延迟时间：0.1s；短路电流密度$J_{sc} = \frac{I_{sc}}{S}$ =16.13 mA/cm²。
计算短路电流密度及转换效率所用面积为掩膜板面积，S=5.059 mm²。

Remark: Forward sweep direction: -0.1V~2.24V, step: 0.02V, delay time: 0.1s. $J_{sc} = \frac{I_{sc}}{S}$ =16.13 mA/cm²
The area used to calculate J_{sc} and Conversion efficiency is determined by mask, S=5.059 mm².

检 验 检 测 结 果
Test Results

序号<br>Clause	检验项目<br>Test item(s)	单位<br>Unit	技术要求<br>Technical requirements	结果<br>Results	单项评价<br>Verdict<br>Pass/Fail
2	电流-电压特性的测量<br>(反扫)<br>Current-voltage characteristic measurement<br>(Reverse Scan)	—	在标准试验条件下（样品温度：25℃±2℃，辐照度：1000W/ m²，标准太阳光谱辐照分布符合 IEC 60904-3 规定），测量样品随负荷变化的电流-电压特性。<br>At STC (module temperature: 25℃±2℃, irradiance: 1000W/m², standard solar spectral irradiance distribution corresponds to IEC60904-3), measure the current-voltage characteristics of the cell with the variation of load.	—	—
2.1	开路电压 Voc<br>Open-circuit voltage, Voc	V	—	2.158	—
2.2	短路电流 Isc<br>Short-circuit current, Isc	mA	—	0.8121	—
2.3	最大功率 Pmax<br>Maximum-power, Pmax	mW	—	1.459	—
2.4	最大功率点电压 Vmp<br>Maximum-power voltage, Vmp	V	—	1.900	—
2.5	最大功率点电流 Imp<br>Maximum-power current, Imp	mA	—	0.7681	—
2.6	填充因子 FF，%<br>Fill factor FF, %	—	—	83.25	—
2.7	转换效率 η，%<br>Conversion efficiency η, %	—	$\eta = \frac{P_{max}}{1000W/m^2 \times S} \times 100\%$<br>S 为掩膜板面积/Area S is determined by mask	28.84	—

备注：反扫扫描方向为 2.24V~-0.1V，步进-0.02V，延迟时间：0.1s；短路电流密度$J_{sc} = \frac{I_{sc}}{S}$ =16.05 mA/cm²，
计算短路电流密度及转换效率所用面积为掩膜板面积，S=5.059 mm²。

Remark: Reverse sweep direction: 2.24V~-0.1 V, step: -0.02V, delay time: 0.1s. $J_{sc} = \frac{I_{sc}}{S}$ =16.05 mA/cm²
The area used to calculate J_{sc} and Conversion efficiency is determined by mask, S=5.059 mm².

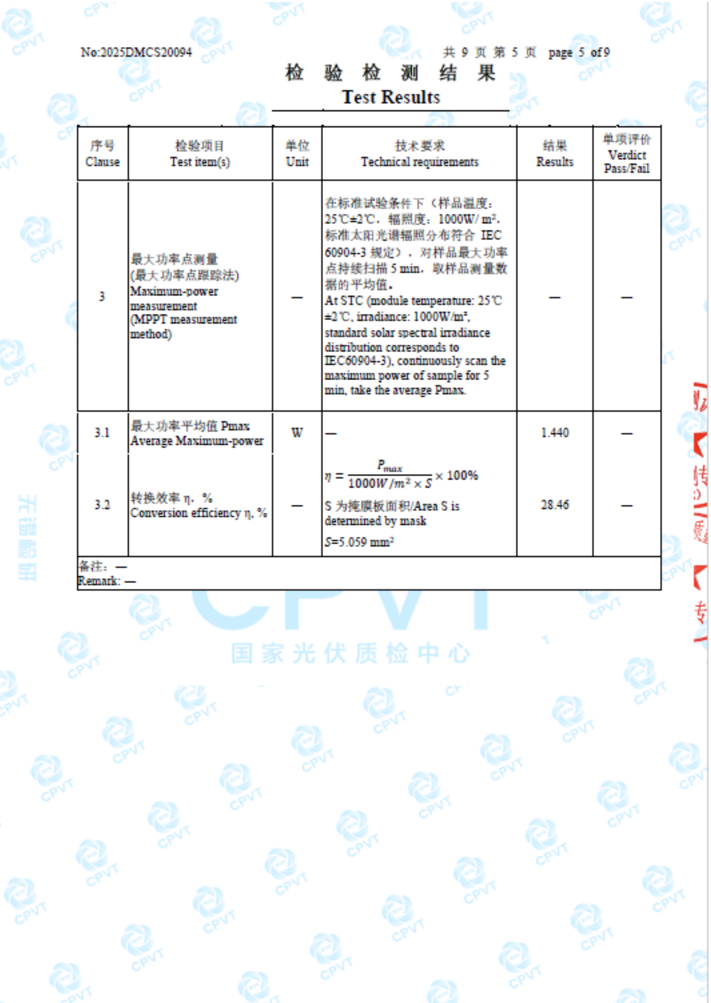

No:2025DMCS20094　　共 9 页 第 5 页 page 5 of 9

检 验 检 测 结 果

Test Results

序号 Clause	检验项目 Test item(s)	单位 Unit	技术要求 Technical requirements	结果 Results	单项评价 Verdict Pass/Fail
3	最大功率点测量 (最大功率点跟踪法) Maximum-power measurement (MPPT measurement method)	—	在标准试验条件下（样品温度：25℃±2℃，辐照度：1000W/ m²，标准太阳光谱辐照分布符合 IEC 60904-3 规定），对样品最大功率点持续扫描 5 min，取样品测量数据的平均值。 At STC (module temperature: 25℃ ±2℃, irradiance: 1000W/m², standard solar spectral irradiance distribution corresponds to IEC60904-3), continuously scan the maximum power of sample for 5 min, take the average Pmax.	—	—
3.1	最大功率平均值 Pmax Average Maximum-power	W	—	1.440	—
3.2	转换效率 η，% Conversion efficiency η, %	—	$\eta = \frac{P_{max}}{1000W/m^2 \times S} \times 100\%$ S 为掩膜板面积/Area S is determined by mask S=5.059 mm²	28.46	—
备注：— Remark: —					

Fig. S27. Efficiency Certification Report. Certification report of a representative all-perovskite tandem solar cell based on SALC-modified Sn-Pb sub-cell by National Center of Inspection on Solar Photovoltaic Products Quality (CPVT).

Table S1. The V_{OC} of single Sn-Pb PSCs and all-perovskite tandem devices.

Samples	Year	S-V_{OC} (V)	V_{OC} deficit (V)	S-PCE (%)	A-V_{OC} (V)	A-PCE (%)	Ref
$FA_{0.6}MA_{0.3}Cs_{0.1}Pb_{0.5}Sn_{0.5}I_3$ (~1.26 eV)	2025	0.88	0.38	23.20	2.17	29.60	10
$Cs_{0.1}FA_{0.6}MA_{0.3}Pb_{0.5}Sn_{0.5}I_3$ (1.25 eV)	2025	0.877	0.373	22.45	2.151	29.16	62
$Cs_{0.1}FA_{0.6}MA_{0.3}Pb_{0.5}Sn_{0.5}I_3$ (1.25 eV)	2025	0.885	0.365	22.88	2.134	28.87	64
$Cs_{0.1}FA_{0.6}MA_{0.3}Pb_{0.5}Sn_{0.5}I_3$ (1.25 eV)	2025	0.89	0.36	23.70	2.13	29.60	65
$FA_{0.6}MA_{0.3}Cs_{0.1}Pb_{0.5}Sn_{0.5}I_3$ (1.25 eV)	2024	0.86	0.39	22.04	2.13	27.17	66
$FA_{0.6}MA_{0.3}Cs_{0.1}Pb_{0.5}Sn_{0.5}I_3$ (1.25 eV)	2024	0.86	0.39	23.0	2.08	27.9	46
$FA_{0.8}Cs_{0.2}Pb_{0.5}Sn_{0.5}I_3$ (~1.27 eV)	2024	0.861	0.409	22.14	2.133	27.41	67
$Cs_{0.05}FA_{0.7}MA_{0.25}Pb_{0.5}Sn_{0.5}I_3$ (1.26 eV)	2024	0.884	0.376	22.7	2.147	27.8	68
$Cs_{0.05}MA_{0.05}FA_{0.9}Pb(I_{0.97}Br_{0.03})_3$ (1.26 eV)	2024	0.877	0.383	23.4	2.16	27.2	69
**$Cs_{0.1}FA_{0.6}MA_{0.3}Pb_{0.5}Sn_{0.5}I_3$ (1.25 eV)**		**0.892**	**0.358**	**22.99**	**2.182**	**29.44**	**This work**

Notes: S-V_{OC} and S-PCE represent the V_{OC} and PCE of single Sn-Pb PSCs. A-V_{OC} and A-PCE represent the V_{OC} and PCE of all-perovskite TSCs.

REFERENCES

1. National Renewable Energy Laboratory. Best Research-Cell Efficiency Chart, https://www.nlr.gov/pv/cell-efficiency [accessed 16 June 2026].

2. T. Leijtens, K. A. Bush, R. Prasanna, M. D. McGehee, Opportunities and challenges for tandem solar cells using metal halide perovskite semiconductors. *Nat. Energy* **3**, 828–838 (2018).

3. R. Wang, T. Huang, J. Xue, J. Tong, K. Zhu, Y. Yang, Prospects for metal halide perovskite-based tandem solar cells. *Nat. Photonics* **15**, 411–425 (2021).

4. K. O. Brinkmann, P. Wang, F. Lang, W. Li, X. Guo, F. Zimmermann, S. Olthof, D. Neher, Y. Hou, M. Stolterfoht, T. Wang, A. B. D. Djurišić, T. Riedl, Perovskite-organic tandem solar cells. *Nat. Rev. Mater.* **9**, 202–217 (2024).

5. H. Chen, A. Maxwell, C. Li, S. Teale, B. Chen, T. Zhu, E. Ugur, G. Harrison, L. Grater, J. Wang, Z. Wang, L. Zeng, S. M. Park, L. Chen, P. Serles, R. A. Awni, B. Subedi, X. Zheng, C. Xiao, N. J. Podraza, T. Filleter, C. Liu, Y. Yang, J. M. Luther, S. D. Wolf, M. G. Kanatzidis, Y. Yan, E. H. Sargent, Regulating surface potential maximizes voltage in all-perovskite tandems. *Nature* **613**, 676–681 (2023).

6. Y. Wang, R. Lin, C. Liu, X. Wang, C. Chosy, Y. Haruta, A. D. Bui, M. Li, H. Sun, X. Zheng, H. Luo, P. Wu, H. Gao, W. Sun, Y. Nie, H. Zhu, K. Zhou, H. T. Nguyen, X. Luo, L. Li, C. Xiao, M. I. Saidaminov, S. D. Stranks, L. Zhang, H. Tan, Homogenized contact in all-perovskite tandems using tailored 2D perovskite. *Nature* **635**, 867–873 (2024).

7. S. Zhou, S. Fu, C. Wang, W. Meng, J. Zhou, Y. Zou, Q. Lin, L. Huang, W. Zhang, G. Zeng, D. Pu, H. Guan, C. Wang, K. Dong, H. Cui, S. Wang, T. Wang, G. Fang, W. Ke, Aspartate all-in-one doping strategy enables efficient all-perovskite tandems. *Nature* **624**, 69–73 (2023).

8. H. Bi, J. Liu, L. Wang, Z. Zhang, G. Kapil, S. R. Sahamir, A. K. Baranwal, Y. Wei, Y. Yang, D. Wang, T. Kitamura, H. Segawa, Q. Shen, S. Hayase, Double side passivation of phenylethyl ammonium iodide for all perovskite tandem solar cell with efficiency of 26.8%. *EcoEnergy* **2**, 369–380 (2024).

9. J. Zhang, X. Liao, W. Li, Y. Tian, Q. Huang, Y. Ji, G. Hu, Q. Du, W. Huang, D. Kim, Y. Cheng, J. Tong, Minimizing tin(II) oxidation using ethylhydrazine oxalate for high-performance all-perovskite tandem solar cells. *J. Semicond.* **46**, 052802 (2025).

10. M. Li, J. Yan, A. Zhang, X. Zhao, X. Yang, S. Yan, N. Ma, T. Ma, D. Luo, Z. Chen, L. Li, X. Li, C. Chen, H. Song, J. Tang, Vacuum-driven precrystallization enables efficient all-perovskite tandem solar cells. *Joule* **9**, 101825 (2025).

11. H. Gao, K. Xiao, R. Lin, S. Zhao, W. Wang, S. Dayneko, C. Duan, C. Ji, H. Sun, A. D. Bui, C. Liu, J. Wen, W. Kong, H. Luo, X. Zheng, Z. Liu, H. Nguyen, J. Xie, L. Li, M. I. Saidaminov, H. Tan, Homogeneous crystallization and buried interface passivation for perovskite tandem solar modules. *Science* **383**, 855–859 (2024).

12. J. Wen, H. Hu, C. Chen, D. P. McMeekin, K. Xiao, R. Lin, Y. Liu, H. J. Snaith, J. Tang, U. W. Paetzold, H. Tan, Present status of and future opportunities for all-perovskite tandem photovoltaics. *Nat. Energy* **10**, 681–696 (2025).

13. S. Hu, J. Wang, P. Zhao, J. Pascual, J. Wang, F. Rombach, A. Dasgupta, W. Liu, M. A. Truong, H. Zhu, M. Kober-Czerny, J. N. Drysdale, J. A. Smith, Z. Yuan, G. J. W. Aalbers, N. R. M. Schipper, J. Yao, K. Nakano, S.-H. Turren-Cruz, A. Dallmann, M. G. Christoforo, J. M. Ball, D. P. McMeekin, K.-A. Zaininger, Z. Liu, N. K. Noel, K. Tajima, W. Chen, M. Ehara, R. A. J. Janssen, A. Wakamiya, H. J. Snaith, Steering perovskite precursor solutions for multijunction photovoltaics. *Nature* **639**, 93–101 (2025).

14. M. A. Green, E. D. Dunlop, M. Yoshita, N. Kopidakis, K. Bothe, G. Siefer, X. Hao, J. Y. Jiang, Solar cell efficiency tables (version 66). *Prog. Photovolt. Res. Appl.* **33**, 795–810 (2025).

15. J. Wen, H. Tan, Present status and future prospects for monolithic all-perovskite tandem solar cells. *Sci. China Mater.* **65**, 3353–3360 (2022).

16. J. Liu, H. Yao, S. Wang, C. Wu, L. Ding, F. Hao, Origins and suppression of Sn(II)/Sn(IV) oxidation in tin halide perovskite solar cells. *Adv. Energy Mater.* **13**, 2300696 (2023).

17. T. Yokoyama, D. H. Cao, C. C. Stoumpos, T.-B. Song, Y. Sato, S. Aramaki, M. G. Kanatzidis, Overcoming short-circuit in lead-free $CH_3NH_3SnI_3$ perovskite solar cells via kinetically controlled gas–solid reaction film fabrication process. *J. Phys. Chem. Lett.* **7**, 776–782 (2016).

18. K. Liang, D. B. Mitzi, M. T. Prikas, Synthesis and characterization of organic-inorganic perovskite thin films prepared using a versatile two-step dipping technique. *Chem. Mater.* **10**, 403–411 (1998).

19. F. Hao, C. C. Stoumpos, P. Guo, N. Zhou, T. J. Marks, R. P. Chang, M. G. Kanatzidis, Solvent-mediated crystallization of $CH_3NH_3SnI_3$ films for heterojunction depleted perovskite solar cells. *J. Am. Chem. Soc.* **137**, 11445–11452 (2015).

20. L. Lanzetta, T. Webb, N. Zibouche, X. Liang, D. Ding, G. Min, R. J. E. Westbrook, B. Gaggio, T. J. Macdonald, M. S. Islam, S. A. Haque, Degradation mechanism of hybrid tin-based perovskite solar cells and the critical role of tin (IV) iodide. *Nat. Commun.* **12**, 2853 (2021).

21. X. Zheng, A. Y. Alsalloum, Y. Hou, E. H. Sargent, O. M. Bakr, All-perovskite tandem solar cells: A roadmap to uniting high efficiency with high stability. *Acc. Mater. Res.* **1**, 63–76 (2020).

22. X. Jiang, Z. Zang, Y. Zhou, H. Li, Q. Wei, Z. Ning, Tin halide perovskite solar cells: An emerging thin-film photovoltaic technology. *Acc. Mater. Res.* **2**, 210–219 (2021).

23. S. Tao, I. Schmidt, G. Brocks, J. Jiang, I. Tranca, K. Meerholz, S. Olthof, Absolute energy level positions in tin- and lead-based halide perovskites. *Nat. Commun.* **10**, 2560 (2019).

24. M. Awais, R. L. Kirsch, V. Yeddu, M. I. Saidaminov, Tin halide perovskites going forward: Frost diagrams offer hints. *ACS Mater. Lett.* **3**, 299–307 (2021).

25. Z. Zhang, Y. Huang, J. Jin, Y. Jiang, Y. Xu, J. Zhu, D. Zhao, Mechanistic understanding of oxidation of tin-based perovskite solar cells and mitigation strategies. *Angew. Chem. Int. Ed. Engl.* **62**, 202308093 (2023).

26. L. Chen, C. Li, Y. Xian, S. Fu, A. Abudulimu, D.-B. Li, J. D. Friedl, Y. Li, S. Neupane, M. S. Tumusange, N. Sun, X. Wang, R. J. Ellingson, M. J. Heben, N. J. Podraza, Z. Song, Y. Yan, Incorporating potassium citrate to improve the performance of tin-lead perovskite solar cells. *Adv. Energy. Mater.* **13**, 2301218 (2023).

27. F. Yang, R. W. MacQueen, D. Menzel, A. Musiienko, A. Al-Ashouri, J. Thiesbrummel, S. Shah, K. Prashanthan, D. Abou-Ras, L. Korte, M. Stolterfoht, D. Neher, I. Levine, H. J. Snaith, S. Albrecht, Rubidium iodide reduces recombination losses in methylammonium-free tin-lead perovskite solar cells. *Adv. Energy Mater.* **13**, 2204339 (2023).

28. P. Wu, J. Wen, Y. Wang, Z. Liu, R. Lin, H. Li, H. Luo, H. Tan, Efficient and thermally stable all-perovskite tandem solar cells using all-FA narrow-bandgap perovskite and metal-oxide-based tunnel junction. *Adv. Energy Mater.* **12**, 2202948 (2022).

29. J. Zhu, Y. Xu, Y. Luo, J. Luo, R. He, C. Wang, Y. Wang, K. Wei, Z. Yi, Z. Gao, J. Wang, J. You, Z. Zhang, H. Lai, S. Ren, X. Liu, C. Xiao, C. Chen, J. Zhang, F. Fu, D. Zhao, Custom-tailored hole transport layer using oxalic acid for high-quality tin-lead perovskites and efficient all-perovskite tandems. *Sci. Adv.* **10**, eadl2063 (2024).

30. C. Liu, R. Lin, Y. Wang, H. Gao, P. Wu, H. Luo, X. Zheng, B. Tang, Z. Huang, H. Sun, S. Zhao, Y. Guo, J. Wen, F. Fan, H. Tan, Efficient all-perovskite tandem solar cells with low-optical-loss carbazolyl interconnecting layers. *Angew. Chem. Int. Ed. Engl.* **62**, e202313374 (2023).

31. X. Sun, H. Wu, Z. Li, R. Zhu, G. Li, Z. Su, J. Zhang, X. Gao, J. Pascual, A. Abate, M. Li, Multifunctional modification of the buried interface in mixed tin-lead perovskite solar cells. *Angew. Chem. Int. Ed. Engl.* **63**, e202409330 (2024).

32. Y. Pan, J. Wang, Z. Sun, J. Zhang, Z. Zhou, C. Shi, S. Liu, F. Ren, R. Chen, Y. Cai, H. Sun, B. Liu, Z. Zhang, Z. Zhao, Z. Cai, X. Qin, Z. Zhao, Y. Ji, N. Li, W. Huang, Z. Liu, W. Chen, Surface chemical polishing and passivation minimize non-radiative recombination for all-perovskite tandem solar cells. *Nat. Commun.* **15**, 7335 (2024).

33. S. Hu, K. Otsuka, R. Murdey, T. Nakamura, M. A. Truong, T. Yamada, T. Handa, K. Matsuda, K. Nakano, A. Sato, K. Marumoto, K. Tajima, Y. Kanemitsu, A. Wakamiya, Optimized carrier extraction at interfaces for 23.6% efficient tin–lead perovskite solar cells. *Energy Environ. Sci.* **15**, 2096–2107 (2022).

34. S. Hu, J. Pascual, W. Liu, T. Funasaki, M. A. Truong, S. Hira, R. Hashimoto, T. Morishita, K. Nakano, K. Tajima, R. Murdey, T. Nakamura, A. Wakamiya, A universal surface treatment for p–i–n perovskite solar cells. *ACS Appl. Mater. Interfaces* **14**, 56290–56297 (2022).

35. S. Hu, P. Zhao, K. Nakano, R. D. J. Oliver, J. Pascual, J. A. Smith, T. Yamada, M. A. Truong, R. Murdey, N. Shioya, T. Hasegawa, M. Ehara, M. B. Johnston, K. Tajima, Y. Kanemitsu, H. J. Snaith, A. Wakamiya, Synergistic surface modification of tin–lead perovskite solar cells. *Adv. Mater.* **35**, e2208320 (2023).

36. L. He, G. Hu, J. Jiang, W. Wei, X. Xue, K. Fan, H. Huang, L. Shen, Highly sensitive tin-lead perovskite photodetectors with over 450 days stability enabled by synergistic engineering for pulse oximetry system. *Adv. Mater.* **35**, e2210016 (2023).

37. R. Sun, P. Wang, L. Zhang, W. Liu, Y. Wen, F. Li, Z. Ge, L. Qiao, T. Wang, T. Ye, P. Ji, X. Yang, Stable high-efficiency monolithic all-perovskite tandem solar cells enabled by a natural reactive oxygen species scavenger. *Energy Environ. Sci.* **17**, 7247–7257 (2024).

38. Q. Chen, J. Luo, R. He, H. Lai, S. Ren, Y. Jiang, Z. Wan, W. Wang, X. Hao, Y. Wang, J. Zhang, I. Constantinou, C. Wang, L. Wu, F. Fu, D. Zhao, Unveiling roles of tin fluoride additives in high-efficiency low-bandgap mixed tin-lead perovskite solar cells. *Adv. Energy Mater.* **11**, 2101045 (2021).

39. R. Lin, K. Xiao, Z. Qin, Q. Han, C. Zhang, M. Wei, M. I. Saidaminov, Y. Gao, J. Xu, M. Xiao, A. Li, J. Zhu, E. H. Sargent, H. Tan, Monolithic all-perovskite tandem solar cells with 24.8% efficiency exploiting comproportionation to suppress Sn (II) oxidation in precursor ink. *Nat. Energy* **4**, 864–873 (2019).

40. K. Xiao, R. Lin, Q. Han, Y. Hou, Z. Qin, H. T. Nguyen, J. Wen, M. Wei, V. Yeddu, M. I. Saidaminov, Y. Gao, X. Luo, Y. Wang, H. Gao, C. Zhang, J. Xu, J. Zhu, E. H. Sargent, H.

Tan, All-perovskite tandem solar cells with 24.2% certified efficiency and area over 1 cm^2 using surface-anchoring zwitterionic antioxidant. *Nat. Energy* **5**, 870–880 (2020).

41. D. Yu, M. Pan, G. Liu, X. Jiang, X. Wen, W. Li, S. Chen, W. Zhou, H. Wang, Y. Lu, M. Ma, Z. Zang, P. Cheng, Q. Ji, F. Zheng, Z. Ning, Electron-withdrawing organic ligand for high-efficiency all-perovskite tandem solar cells. *Nat. Energy* **9**, 298–307 (2024).

42. C. Li, Y. Pan, J. Hu, S. Qiu, C. Zhang, Y. Yang, S. Chen, X. Liu, C. J. Brabec, M. K. Nazeeruddin, Y. Mai, F. Guo, Vertically Aligned 2D/3D Pb–Sn perovskites with enhanced charge extraction and suppressed phase segregation for efficient printable solar cells. *ACS Energy Lett.* **5**, 1386–1395 (2020).

43. R. Lin, J. Xu, M. Wei, Y. Wang, Z. Qin, Z. Liu, J. Wu, K. Xiao, B. Chen, S. M. Park, G. Chen, H. R. Atapattu, K. R. Graham, J. Xu, J. Zhu, L. Li, C. Zhang, E. H. Sargent, H. Tan, All-perovskite tandem solar cells with improved grain surface passivation. *Nature* **603**, 73–78 (2022).

44. W. Liao, D. Zhao, Y. Yu, N. Shrestha, K. Ghimire, C. R. Grice, C. Wang, Y. Xiao, A. J. Cimaroli, R. J. Ellingson, N. J. Podraza, K. Zhu, R. Xiong, Y. Yan, Fabrication of efficient low-bandgap perovskite solar cells by combining formamidinium tin iodide with methylammonium lead iodide. *J. Am. Chem. Soc.* **138**, 12360–12363 (2016).

45. J. Tong, Q. Jiang, A. J. Ferguson, A. F. Palmstrom, X. Wang, J. Hao, S. P. Dunfield, A. E. Louks, S. P. Harvey, C. Li, H. Lu, R. M. France, S. A. Johnson, F. Zhang, M. Yang, J. F. Geisz, M. D. McGehee, M. C. Beard, Y. Yan, D. Kuciauskas, J. J. Berry, K. Zhu, Carrier control in Sn-Pb perovskites via 2D cation engineering for all-perovskite tandem solar cells with improved efficiency and stability. *Nat. Energy* **7**, 642–651 (2022).

46. Y. Bai, R. Tian, K. Sun, C. Liu, X. Lang, M. Yang, Y. Meng, C. Xiao, Y. Wang, X. Lu, J. Wang, H. Pan, Z. Song, S. Zhou, Z. Ge, Decoupling light- and oxygen-induced degradation mechanisms of Sn–Pb perovskites in all perovskite tandem solar cells. *Energy Environ. Sci.* **17**, 8557–8569 (2024).

47. J. Xi, M. A. Loi, The fascinating properties of tin-alloyed halide perovskites. *ACS Energy Lett.* **6**, 1803–1810 (2021).

48. J. Seo, T. Song, S. Rasool, S. Park, J. Y. Kim, An overview of lead, tin, and mixed tin–lead-based ABI_3 perovskite solar cells. *Adv. Energy Sustain. Res.* **4**, 2200160 (2023).

49. V. J.-Y. Lim, A. M. Ulatowski, C. Kamaraki, M. T. Klug, L. Miranda Perez, M. B. Johnston, L. M. Herz, Air-degradation mechanisms in mixed lead-tin halide perovskites for solar cells. *Adv. Energy Mater.* **13**, 2200847 (2023).

50. A. R. Bowman, M. T. Klug, T. A. S. Doherty, M. D. Farrar, S. P. Senanayak, B. Wenger, G. Divitini, E. P. Booker, Z. Andaji-Garmaroudi, S. Macpherson, E. Ruggeri, H. Sirringhaus, H. J. Snaith, S. D. Stranks, Microsecond carrier lifetimes, controlled p-doping, and enhanced air stability in low-bandgap metal halide perovskites. *ACS Energy Lett.* **4**, 2301–2307 (2019).

51. W. Zhang, H. Yuan, X. Li, X. Guo, C. Lu, A. Liu, H. Yang, L. Xu, X. Shi, Z. Fang, H. Yang, Y. Cheng, J. Fang, Component distribution regulation in Sn-Pb perovskite solar cells through selective molecular interaction. *Adv. Mater.* **35**, e2303674 (2023).

52. R. G. Acres, V. Feyer, N. Tsud, E. Carlino, K. C. Prince, Mechanisms of aggregation of cysteine functionalized gold nanoparticles. *J. Phys. Chem. C* **118**, 10481–10487 (2014).

53. T. A. A. Oliver, G. A. King, M. G. D. Nix, M. N. R. Ashfold, Ultraviolet photodissociation dynamics of 2-methyl, 3-furanthiol: Tuning π-conjugation in sulfur substituted heterocycles. *J. Phys. Chem. A* **114**, 1338–1346 (2010).

54. J. Poater, M. Duran, M. Solà, B. Silvi, Theoretical evaluation of electron delocalization in aromatic molecules by means of atoms in molecules (AIM) and electron localization function (ELF) topological approaches. *Chem. Rev.* **105**, 3911–3947 (2005).

55. B. Li, B. Chang, L. Pan, Z. Li, L. Fu, Z. He, L. Yin, Tin-based defects and passivation strategies in tin-related perovskite solar cells. *ACS Energy Lett.* **5**, 3752–3772 (2020).

56. L. Xie, S. Du, J. Li, C. Liu, Z. Pu, X. Tong, J. Liu, Y. Wang, Y. Meng, M. Yang, W. Li, Z. Ge, Molecular dipole engineering-assisted strain release for mechanically robust flexible perovskite solar cells. *Energy Environ. Sci.* **16**, 5423–5433 (2023).

57. C. Liu, Y. Yang, H. Chen, J. Xu, A. Liu, A. S. R. Bati, H. Zhu, L. Grater, S. S. Hadkes, C. Huang, V. K. Sangwan, T. Cai, D. Shin, L. X. Chen, M. C. Hersaml, C. A. Mirkin, B. Chen, M. G. Kanatzidisl, E. H. Sargent, Bimolecularly passivated interface enables efficient and stable inverted perovskite solar cells. *Science* **382**, 810–815 (2023).

58. J. Zhou, S. Fu, S. Zhou, L. Huang, C. Wang, H. Guan, D. Pu, H. Cui, C. Wang, T. Wang, W. Meng, G. Fang, W. Ke, Mixed tin-lead perovskites with balanced crystallization and oxidation barrier for all-perovskite tandem solar cells. *Nat. Commun.* **15**, 2324 (2024).

59. Z. Wang, J. Liu, B. Zhang, L. Sun, L. Cong, L. Li, A. Mauger, C. M. Julien, H. Xie, H. Sun, Modulating molecular orbital energy level of lithium polysulfide for high-rate and long-life lithium-sulfur batteries. *Energy Storage Mater.* **24**, 373–378 (2020).

60. S. Chen, X. Xiao, H. Gu, J. Huang, Iodine reduction for reproducible and high-performance perovskite solar cells and modules. *Sci. Adv.* **7**, eabe8130 (2021).

61. X. Cao, J. Li, H. Dong, P. Li, Q. Fan, R. Xu, H. Li, G. Zhou, Z. Wu, Stability improvement of tin-based halide perovskite by precursor-solution regulation with dual-functional reagents. *Adv. Funct. Mater.* **31**, 2104344 (2021).

62. X. Yang, T. Ma, H. Hu, W. Ye, X. Li, M. Li, A. Zhang, C. Ge, X. Sun, Y. Zhu, S. Yan, J. Yan, Y. Zhou, Z. Li, C. Chen, H. Song, J. Tang, Understanding and manipulating the crystallization of Sn-Pb perovskites for efficient all-perovskite tandem solar cells. *Nat. Photonics* **19**, 426–433 (2025).

63. D. Luo, W. Yang, Z. Wang, A. Sadhanala, Q. Hu, R. Su, R. Shivanna, G. F. Trindades, J. F. Watts, Z. Xu, T. Liu, K. Chen, F. Ye, P. Wu, L. Zhao, J. Wu, Y. Tu, Y. Zhang, X. Yang, W. Zhang, R. H. Friend, Q. Gong, H. J. Snaith, R. Zhu, Enhanced photovoltage for inverted planar heterojunction perovskite solar cells. *Science* **360**, 1442–1446 (2018).

64. Y. Bai, Y. Meng, M. Yang, R. Tian, J. Wang, B. Jiao, H. Pan, J. Gao, Y. Wang, K. Sun, S. Zhou, X. Lu, Z. Song, C. Liu, Z. Ge, Lattice stabilization and strain homogenization in Sn-Pb bottom subcells enable stable all-perovskite tandems solar cells. *Nat. Commun.* **16**, 7344 (2025).

65. J. Wang, S. Hu, H. Zhu, S. Liu, Z. Zhang, R. Chen, J. Wang, C. Shi, J. Zhang, W. Liu, X. Lei, B. Liu, Y. Pan, F. Ren, H. Raza, Q. Zhou, S. Li, L. Qiu, G. Zheng, X. Qin, Z. Zhao, S. Yang, N. Li, J. Li, A. Wakamiya, Z. Liu, H. J. Snaith, W. Chen, Mercapto-functionalized scaffold improves perovskite buried interfaces for tandem photovoltaics. *Nat. Commun.* **16**, 4917 (2025).

66. G. Zeng, W. Chen, H. Guan, W. Meng, H. Cui, S. Zhou, L. Huang, D. Pu, H. Fang, H. Liu, S. Hou, T. Yu, G. Fang, W. Ke, Trapping tetravalent tin and protecting stannous in tin-lead mixed perovskites for efficient all-perovskite tandem solar cells. *Adv. Funct. Mater.* **35**, 2412458 (2024).

67. J. Wang, Y. Pan, Z. Zhou, Q. Zhou, S. Liu, J. Zhang, C. Shi, R. Chen, Z. Zhao, Z. Cai, X. Qin, Z. Zhao, Z. Yang, Z. Liu, W. Chen, Bimolecular crystallization modulation boosts the efficiency and stability of methylammonium-free tin–lead perovskite and all-perovskite tandem solar cells. *Adv. Energy Mater.* **14**, 2402171 (2024).

68. S. Fu, N. Sun, Y. Xian, L. Chen, Y. Li, C. Li, A. Abudulimu, P. N. Kaluarachchi, Z. Huang, X. Wang, K. Dolia, D. S. Ginger, M. J. Heben, R. J. Ellingson, B. Chen, E. H. Sargent, Z. Song, Y. Yan, Suppressed deprotonation enables a durable buried interface in tin-lead perovskite for all-perovskite tandem solar cells. *Joule* **8**, 2220–2237 (2024).

69. S. Tan, C. Li, C. Peng, W. Yan, H. Bu, H. Jiang, F. Yue, L. Zhang, H. Gao, Z. Zhou, Sustainable thermal regulation improves stability and efficiency in all-perovskite tandem solar cells. *Nat. Commun.* **15**, 4136 (2024).
